# Climatic comparison of surface urban heat island using satellite remote sensing in Tehran and suburbs

**DOI:** 10.1038/s41598-023-50757-2

**Published:** 2024-01-05

**Authors:** Motahhareh Zargari, Abbas Mofidi, Alireza Entezari, Mohammad Baaghideh

**Affiliations:** 1 Hakim Sabzevari University, Sabzevar, Iran; 2 Ferdowsi University of Mashhad, Mashhad, Iran

**Keywords:** Climate sciences, Environmental sciences

## Abstract

In this study, we aim to compare the climatic conditions of Surface Urban Heat Island (SUHI) in Tehran and its suburbs using day/night time data from three satellites. A high-resolution Land Surface Temperature (LST) data from MODIS Aqua, Sentinel-3, and Landsat 8 were selected to facilitate this study. The highest values of LST/UHI are observed in downtown Tehran and suburban areas at night. The temperature difference also shows an increase at night in Tehran and the western suburbs, while it decreases during the day. When comparing LST/UHI with altitude in different directions, it is found that urban areas and the south, southeast, southwest, and west suburban areas experience higher temperatures at night. MODIS LST products are more appropriate for checking nighttime SUHI in Tehran's Great area in comparison to other products. Moran's I indicates that the highest positive values occur during seasonal and annual periods at night. The Getis index demonstrates a consistent pattern across all seasons, and this trend persists throughout the year. The seasonal and annual UHI difference between Tehran and its suburbs is 5 °C. The LST diagram reveals that higher temperatures occur during warm months. The temporal NDVI distribution indicates lower NDVI values from June to February and summer to winter. The spatial distribution shows that due to the lack of NDVI index in urban areas, LST/UHI values are higher at night in Tehran compared to the suburbs. UHI is not limited to urban areas but has also spread beyond the city borders. As a result, the highest UHI values are found in downtown Tehran and its southeast, south, southwest, and west suburbs.

## Introduction

The Surface Urban Heat Island (SUHI) is a micro-climate phenomenon that occurs in urban environments, where the air temperatures in urbanized areas are significantly higher than those in outlying areas^1–4^. In fact, rapid urbanization become one of the most important global issues in the twenty-first century^5–8^,which the SUHI being one of the most significant manifestations resulting from it. Reports suggest that by 2030, more than 1.2 million km^2^ of land will be converted into urban areas, which is twice the amount compared to 2000. Half of this expansion is expected to occur in Asian cities^9^, resulting in over 2.5 billion people residing in urban areas. The United Nations predicts that by 2050, 68% of the world's population will live in urban areas, with most residing in Asia and Africa^10^. Tehran megalopolis, the largest and most populous city, in west Asia, has experienced rapid urbanization and expansion over the past few decades^11,12^. Nowadays, the city environment is filled with heat-absorbing surfaces, such as built areas replacing natural land cover causing man-made surfaces to become warmer by absorbing heat^13,14^. Tehran, with a residential area of 266 km^2^ and activity zones (commercial, administrative, and industrial) covering 182 km^2^, is a large city with a high amount of built-up areas. As a result, the average surface temperature in residential, industrial, and barren areas is much higher^15^. 85 percent of Tehran's land cover consists of constructed areas^16^. On the other hand, the high density of building and constructed areas is one of the main reasons for the SUHI in Tehran^17^.

The SUHI in Tehran has been increasing over the last three decades, with an average value of 2.02 °C^18^. Tehran's SUHI has expanded towards the western and southwestern regions, with the main focus on the western, southwestern, central, and southern areas. This expansion is accompanied by a sharp reduction in vegetation and a significant increase in industrial-workshop land use. The area of green spaces per capita is less than 10 m^2^ in eight districts of Tehran^19^. Therefore, these factors not only result in higher temperatures during the day but also prevent from cooling down at night. Warmer nights are particularly concerning as they contribute to the occurrence of extreme heat events, which can cause major problems in key parts of the metropolis^20^. The enhanced Urban Heat Island (UHI) and growth will have a negative impact on natural ecosystems, resulting in significant changes in land use, climate systems, biological diversity, and impervious surfaces^21–25^.

Tehran SUHI can be controlled through proper city design and vegetation management. Managing the heat flow at the downtown requires urban planning and remote sensing in design and implementation^26^. Furthermore, the area of built-up land in Tehran has increased by 88% from 1985 to 2019, and this trend is expected to continue in the future. The SUHI has also increased from 0.01 in 1985 to 0.34 in 2019. Nevertheless, it is projected to reach 0.38, 0.45, and 0.51 in 2026, 2032, and 2038 respectively^27^. Additionally, temporal changes in the UHI ratio in Tehran during 2017 revealed that the intensity of the UHI was higher than the average (approximately 0.067) because of vegetation, climate conditions, and air pollution^28^.

Hashemi et al.^29^. demonstrated that Tehran's SUHI exhibits temporal–spatial diurnal and seasonal variation. With regard to thermal characteristics, different land cover thermal properties, albedo, and altitude are essential factors contributing to daily changes in SUHI within this city; whereas vegetation and albedo are critical factors affecting seasonal changes in SUHI. It is worth noting that there have been significant spatial changes in UHI within the city over the last two decades, with the UHI center at Mehrabad airport expanding towards the west and southwest regions of Tehran in recent years^30^. However, there is a lack of a coherent plan to decentralize urban facilities from the central parts of the city. This has resulted in unplanned urban development in Tehran. The density and diversity of neighborhoods and the design of road networks can have various economic, social, and environmental effects. Furthermore, the lack of vegetation in the city compared to the surrounding rural environment leads to an increase in man-made surface temperature and a decrease in evaporation^26^. However, it faces a severe lack of observation data for studying urban climates. This issue has led to a significant increase in urban studies based on satellite data for Tehran due to the development of satellite products, their variety, and ease of access^31–33^. The use of satellite sensor data with high spatial–temporal resolution presents an excellent opportunity to overcome current limitations in ground meteorological data, especially in times and places where observations based on meteorological stations are insufficient or incomplete^34^.

Despite conducting comprehensive surveys, a detailed study on some of the crucial features of Tehran's UHI has not been done yet. The intensity and temperature difference between the city and its suburbs are still unknown. Therefore, remote sensing is a useful tool to investigate the urban climate. Remote sensing provide valuable information for creating thermal maps and estimating radiation energy due to their wide and timely coverage. Several satellites such as MODIS Aqua^35^, Sentinel-3^36^, and Landsat 8^37–43^. have been utilized to monitor and evaluate UHI. Numerous research studies have been conducted on UHI in Tehran using remote sensing techniques. Previous studies have primarily focused on modeling and spatial–temporal analysis of the UHI in Tehran^44,45^, and no previous domestic or foreign research has explored this topic, making this study innovative. However, this particular study is significant as it examines both daytime and nighttime products from various satellites. The present study aims to compare climatic SUHI in three-satellite using day/night time products in Tehran and suburbs. By analyzing combined satellite data from both day and night during the summer, the performance of each satellite is evaluated, and the best one is selected for climate studies.

## Methods

### Study area

**Figure 1:**
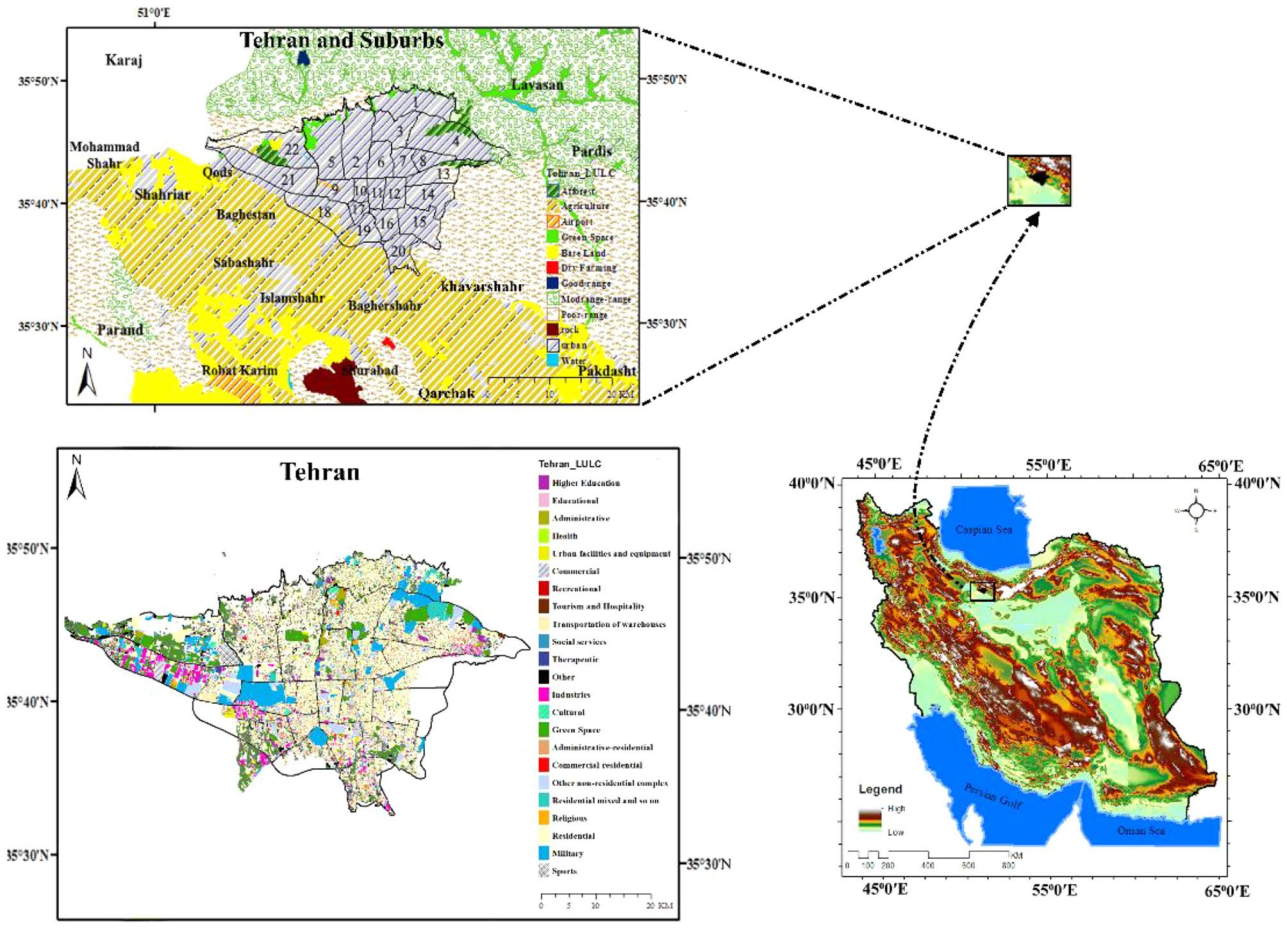
The location of 22 districts in Tehran, including both urban and suburban areas, with regards to Land Cover/Land Use (LC/LU). The data first were prepared using digital elevation model (DEM). URL link: https://data.nextgis.com/en/region/IR/dem and then by Esri Land Cover/Land Use. URL link: https://livingatlas.arcgis.com/landcover. The maps were generated in ArcGIS. ESRI ArcGIS Desktop. Release 10. Redlands, CA: Environmental Systems Research Institute. Version number: ArcGIS 10.8. URL link: https://www.esri.com (2020), and also in paint.net. Version number: paint.net 4.3.11. URL link: https://www.getpaint.net (2022).

Iran is the 18th largest country in the world, covering an area of 1,648,195 km^2^. It is also the most populous country in the Middle East, with a population of 83.70 million^46,47^. As shown in Fig. 1, Iran is situated between latitudes 25°3′ and 39°47′N and longitudes 44°5′ and 63°18′E in Southwest Asia^48^. The majority of its regions have an arid or semi-arid climate^49^. Tehran, with an area of 730 km^2^, is the largest city^50,51^. It is also one of the largest metropolises in the Middle East^52^. The average altitude of Tehran is around 1200 m above sea level. The city covers only about 2.1% of Iran’s total area^53^ and lies between latitudes 35°34′ N to 35°59′ N and longitudes 51°5′E to 51°53′E^54^. This city is surrounded by mountain ranges on its northern and northeastern sides and also lies in a valley with an altitude ranging from 1000 to1800 m above sea level^55^. The average temperature in Tehran is around 19.1 °C^56^.

### Data

Remote sensing instruments can be placed on various platforms to observe and capture images of targets. While ground-based and aircraft platforms can be used, satellites play a significant role in providing remote sensing imagery that is commonly used today. Satellites possess unique characteristics that make them particularly useful for remote sensing of the land surface. In this study, the high-resolution satellite sensors data from MODIS Aqua, Sentinel-3, and Landsat 8 (Table 1) were utilized due to the lack of observational data. This was done in order to examine the variation of the SUHI in Tehran.

**Table 1:** The fundamental characteristics of the selected satellites for studying SUHI in Tehran.

Raw	Satellite type	Spatial resolution	Bands	Temporal resolution	Local time
1	MODIS Aqua	1 km	36	2 days	01:30 a.m. 01:30 p.m.
2	Sentinel-3	1 km	11	2 days	B: 06:25:56 a.m. A: 06:22:17 p.m.
3	Landsat 8	100 m	11	16 days	10 a.m.

The study aimed to analyze SUHI on June 22, 2020. This date was specifically chosen due to favorable conditions such as minimal cloud coverage and extensive surface data coverage across all three sensors. Therefore, a high-resolution Land Surface Temperature (LST) data for the selected day was obtained from three different satellites, with detailed descriptions for each satellite provided separately in Table 2. In this regard, data from two databases, Earth data and Google Earth Engine (GEE), were used for MODIS Aqua. Sentinel-3 from Copernicus and Landsat 8 from Earth Explorer were utilized.

**Table 2:** LST databases in various satellite sensors.

	Raw	Data	Satellite sensors	Launch	Address	Format
LST	1	Earth data	MODIS Aqua	National Aeronautics and Space Administration (NASA)	https://appeears.earthdatacloud.nasa.gov/	TIF
	2	Google Earth Engine (GEE)		Google	https://earthengine.google.com/	
	3	Copernicus	Sentinel-3	European Space Agency (ESA)	https://scihub.copernicus.eu/	
	4	Earth explorer	Landsat 8	United States Geological Survey (USGS)	https://earthexplorer.usgs.gov/	

#### MODIS Aqua

This study utilizes MODIS Aqua data with a 1 km spatial resolution, collected during both day and night. The Aqua satellite passes from south to north at around 1:30 a.m. and 1:30 p.m., making it the afternoon satellite^57^. The MODIS Aqua version 6.1 includes a set of LST and Emissivity (E) products, which are used in this research with the MYD11 algorithm. It is important to note that the MYD11 LST uses the split-window (SW) algorithm, whereas the MYD21 LST algorithm is founded on the ASTER Temperature Emissivity Separation (TES) technique. Both algorithms are appropriate for this study area as they exhibit minimal daytime and nighttime errors. However, MYD11 was selected to demonstrate superior LST detection in our region. Recent evaluations have demonstrated that the TES algorithm exhibits greater accuracy in estimating LST in subtropical, hot, and dry climates^58^. However, SWA showed better performance over bare soils^59^. Thus, for the analysis of the MODIS Aqua, MYD11A1 and MYD13A3 products were used (Table 3).

**Table 3:** The data in MODIS Aqua.

Raw	Satellite sensor	Algorithm	Layers	Products
1	MODIS Aqua	SW	LST_Night_1km	Aqua MODIS Land Surface Temperature & Emissivity (LST & E): MYD11A1,061, 1000 m, Daily (2002-07-04 to present)
			LST_Day_1km	
2			_1_km_monthly_NDVI	Aqua MODIS Vegetation Indices (NDVI & EVI): MYD13A3, 061, 1000 m, Monthly (2002-07-01 to present)

#### Sentinel-3

The Sentinel-3 satellites are equipped with a high-resolution scanner that enables the estimation of LST. The Sea and Land Surface Temperature Radiometer (SLSTR) is a temperature radiometer that has dual-view capability. The Sentinel-3 comprises three versatile satellites, namely Sentinel-3A, Sentinel-3B, and Sentinel-3C, which have a combined mission for both ocean and land. The SLSTR level-2 product, SL_2_SLT, represents LST in this satellite^60^. The algorithm employed for estimating LST in the SLSTR sensor relies on the SW, which utilizes Land Surface Emissivity (LSE) for computations^61^. According to reports, the accuracy of the LST algorithm with 1km is significantly higher during nighttime than daytime^62^. The LST data from the Sentinel A satellite at 6:22 p.m. (local time) and Sentinel B data at 6:25 a.m. were utilized as the day and night time data, respectively (Table 4). It is important to note that the S7, S8, and S9 bands with a spatial resolution of 1 km were used in generating the LST data (Appendix A).

**Table 4:** The data in Sentinel-3.

Raw	Satellite sensor	Algorithm	Layers	Instrument	product type	Product level	Orbit direction (start)
1	Sentinel-3	SW	A (night)	SLSTR	SL_2_LST___	L2	Ascending
2			B (day)				Descending

#### Landsat 8

Landsat 8 is equipped with a thermal infrared sensor (TIRS) that is used to calculate LST. This approach is particularly useful for obtaining LST information on a regional and global scale, as the sensor detects the highest amount of energy emitted directly from the land surface^63^. LST can be calculated using various algorithms, including the single-window (SW), SW, and single-channel (SC) algorithms. In this study, the SC algorithm was employed to calculate LST in the study area. This algorithm was chosen due to its utilization of atmospheric and water vapor transmission coefficients as inputs, which are more accurate than those used in other algorithms in arid regions. The SC algorithm has been found to exhibit the highest accuracy in estimating LST, owing to its use of the atmospheric transmission coefficient as an input^64^. For this study, Landsat 8 Collection-1 Level-1 data products were utilized to estimate LST (Table 5). The process involved analyzing the thermal bands 10 and 11 within the spectral range, with a spatial resolution of 100 meters^65^.

**Table 5:** The data in Landsat 8.

Raw	Satellite sensor	Algorithm	Day/night indicator	Sensor identifier	Data type	Data level	Station identifier
1	Landsat 8	SC	Day	OLI_TIRS	OLI_TIRS_L1TP	L1	LGN

### Method

#### Calculate LST in three satellites

The MODIS and Sentinel-3 corrected LST data have a horizontal resolution of 1 km for both day and night hours. After receiving the images, initial processing of the LST was conducted on various satellites. This involved a series of steps such as Project Raster, scaling, and converting Kelvin to Celsius for the MYD11A1 Aqua satellite product for both night and day layers in the ArcGIS environment (Eq. 1). The same process was applied to Sentinel-3 for layers A and B for both night and day in the SNAP (Eq. 2).

1 $${\text{LST }} = \, \left( {{\text{LST image in Kelvin }}* \, 0.0{2}} \right) \, {-}{ 273}.{15}$$

2 $${\text{LST }} = {\text{ LST image in Kelvin }}{-}{ 273}.{15}$$

However, the estimation process for LST requires image pre-processing, processing, and radiometry calibration in Landsat 8 (Appendix B). Subsequently, LST maps were created for both day and night using all satellites except Landsat 8 due to the absence of night data.

#### Calculate UHI in three satellites

First, the corrected LST image in “Calculate LST in three satellites” section was separately analyzed for each satellite for the selected day, June 22, 2020, to extract the minimum and maximum temperatures. The minimum and maximum temperatures were extracted on the LST image. Then, the SUHI of Tehran was processed using SUHI index in Eq. (3)^66^ in ArcGIS and SNAP. The use of this index enables the normalization of the LST values between 0 and 1 for each pixel in an image.

3 $${{\text{SUHI}}}_{{\text{index}}}={({\text{LST}}}_{{\text{i}}}-{{\text{LST}}}_{{\text{min}}})/{({\text{LST}}}_{{\text{max}}}-{{\text{LST}}}_{{\text{min}}})$$

LSTi represents the LST of a pixel in a specific image, while LSTmax and LSTmin denote the maximum and minimum values of LST, respectively, for the same image as that of LSTi. Also, the LST/SUHI spatial distribution of MODIS was calculated in GEE environment for 2020 and analyzed in GIS. Additionally, the temperature difference between the city and its suburbs was calculated and depicted as a separate map for day and night across all three satellites in Qgis. Elevation is a crucial factor in investigating LST and SUHI. in three satellites. So the relationship between altitude and LST/UHI was drawn in QGIS. This approach provided a comprehensive view of the formation of this phenomenon in Tehran. By examining LST and SUHI changes separately for day and night in different directions. In the following, we focus solely on MODIS Aqua for analyzing LST and UHI in Tehran.

#### Moran’s I and Getis-Ord Gi* in MODIS Aqua

The Global Moran's statistic^67^ is typically used to measure similar values at neighboring locations as an index of spatial dependence. This statistic has been utilized in the study area to estimate the spatial characteristics and describe the spatial distribution of LST. The index was calculated seasonally and annually for both day and night in ArcGIS to determine whether LST exhibited a clustered or scattered pattern (Appendix C). Although Moran is a useful tool for measuring similarity, the family of Moran indices (both local and global) fails to differentiate between hot and cold spots. In contrast, the Getis-Ord Gi* index is better suited for identifying such spots as it can pinpoint unsafe areas on a global scale and identify cluster structures with high or low concentrations among local observations. The Getis-Ord Gi* statistic, developed by Getis and Ord^68,69^, is a measure that focuses on identifying hot and cold spots. Unlike Moran's statistic, which classifies clusters, the Getis-Ord Gi* statistic deals with identifying clusters directly.

The criterion for identifying a statistically significant hot-spot (or cold-spot) is that the feature/area should have the highest (or lowest) value, while being surrounded by other features/areas with similar LST values^69^. The analyses for this study were conducted using the Hot-spot Analysis tool (Getis-Ord Gi) available in ArcGIS ESRI environment^70^. The method was performed on the averages of nighttime summer LST dataset from MODIS Aqua imageries, previously converted to a spatial point object. Considering the MODIS resampling carried out by NASA (1 km spatial resolution), the horizontal distance of each LST point object was 1 km. Getis-Ord Gi* statistic method was focused on the calculation for each LST point taken in the context of neighboring points. This index was calculated seasonally and annually for day and night in GIS environment (Appendix D).

Getis-Ord Gi* application performed in GIS requires an appropriate bandwidth to be selected (expressed in meters) in order to perform a reliable hot-spot analysis. The methodological approach suggests the use of metrics such as inverse Euclidean distance and neighbor maximum distance by considering only the eight neighboring pixels^71^. This approach was applied in the present study for hot-spot detection and the corresponding bandwidth value was about 42.43 m. The output of the Gi* statistic also provides measures of statistical significance for each feature/area: the probability (Gi* p-value) and standard deviation (Gi* z-score) values. Gi* z-score is a measure of how much the features/areas are clustered, and the Gi* p-value is the probability that the hot-spot patterns found are only due to a random spatial process. Strictly speaking, a high z-score and a low p-value indicate a significant hot-spot. A low negative z-score and a small p-value indicates a significant cold-spot. The higher (or lower) the z-score, the more intense is the clustering. Thus, three macro groups of thermal patterns were identified: (1) cool-spot: statistically significant clustering of low LST values (Gi* z-score <  − 1.65); (2) hot-spot: statistically significant clustering of high LST values (Gi* z-score > 1.65); 3. other areas with no significant spatial correlation (− 1.65 < Gi* z-score < 1.65).

Confidence level identifies the statistical significance of hot- and cool-spots at thresholds of 90%, 95%, and 99%. Therefore, in the Florentine metropolitan area, hot-spot were categorized by 3 × 2 progressive groups based on confidence level thresholds^72^: “Cold-spot99 (cold-spot LEVEL-3)”, “Cold-spot95 (cold-spot LEVEL-2)”, “Cold-spot90 (cold-spot LEVEL-1)”, “Hot-spot90 (hot-spot LEVEL-1)”, “Hot-spot95 (hot-spot LEVEL-2), “Hot-spot99 (hot-spot LEVEL-3)”. Table 6 shows the hot- and cold-spot classification based on Getis-Ord Gi* approach.

**Table 6:** Hot- and cold- spot classification by applying Getis-Ord Gi* approach.

Gi*Hot-spot classes	Confidence levels	Probability (Gi* p-value)	Standard deviation (Gi* z-Score)
Cold-spot 99 (Level-3)	99%	< 0.01	< − 2.58
Cold-spot 95 (Level-2)	95%	< 0.05	< − 1.96
Cold-spot 90 (Level-1)	90%	< 0.10	< − 1.65
Other areas	Not significant	0	− 1.65 < z-score < 1.65
Hotspot 90 (Level-1)	90%	< 0.10	> 1.65
Hot-spot 95 (Level-2)	95%	< 0.05	> 1.96
Hot-spot 99 (Level-3)	99%	< 0.01	> 2.58

The highest standard deviation (Gi* z-score > 2.58 or Gi* z-score <  − 2.58) and the lowest probability (Gi* p-value < 0.01) classes defined areas with extreme values (LEVEL-3) where the highest or lowest LST values were clustered with 99% confidence level. An Inverse Distance Weighted (IDW) interpolation technique was applied on the Getis-Ord Gi* resulting points maps, in this way obtaining hot- and cool-spot raster maps with MODIS Aqua grid resolution. This method is suitable to interpolate a raster surface from dense data by using a specific search radius^73^. These outputs were vectorized to obtain hot- and cool-spots as a polygonal feature layer.

According to hot and cold spots, UHI difference was calculated between urban and suburban areas both seasonally and annually in QGIS.

Additionally, the per capita green space in Tehran was assessed by utilizing the vegetation index, which is based on the unique reflection pattern of plants. These reflection characteristics are utilized to create plant indices, as described by Huete^74^. One such indicator is the Normalized Difference Vegetation Index (NDVI) is considered as land use land cover indicator to quantitatively explore the relationship between the thermal environment and urban expansion^75,76^, and to investigate the greening effect on LST changes^77–79^. In this study, NDVI is derived from MODIS Aqua (MYD13A3) monthly product by using high-resolution (1 km) remote sensing imagery of the summer nighttime in ArcGIS.

## Result

### The spatial investigation of LST in three-satellite sensors

LST is a crucial indicator used to measure the land environment, with significant impacts on the ecological system and human life^80–82^. Therefore, LST can be used to identify UHI. As the warm season, particularly summer, has the highest frequency and intensity of UHI occurrence, this study focuses on summer in Tehran. During the day, MODIS Aqua, Sentinel-3, and Landsat 8 demonstrate that the northern suburbs and northern to central urban areas have the lowest LST spatial distribution. Despite this, it is evident that the other rural areas exhibit the highest LST. Conversely, most urban areas and northern suburbs display lower LST values compared to other regions, even though; the eastern, western, and southern suburbs record the highest LST values, which are particularly prominent in the MODIS Aqua images. Temperature fluctuations are frequently observed across these three satellite images, with a consistent pattern evident (a, c, e). However, temperature conditions vary during nighttime. The urban areas show an increase in LST in both satellites, particularly in MODIS Aqua. The whole city plus suburbs (except north suburbs) show the highest LST values in Sentenil-3 which are much more distinguishable in central urban areas in MODIS Aqua. This clearly shows that LST is higher in urban areas rather than rural areas. The lowest LST is observed in the northern suburbs. Moving towards lower regions, LST gradually increases in the urban and suburbs areas, with the highest LST recorded in the city and then in other suburbs (except northern suburbs). Notably, night-time LST values are higher in Tehran than its suburbs, especially in MODIS Aqua (b, d).

**Figure 2:**
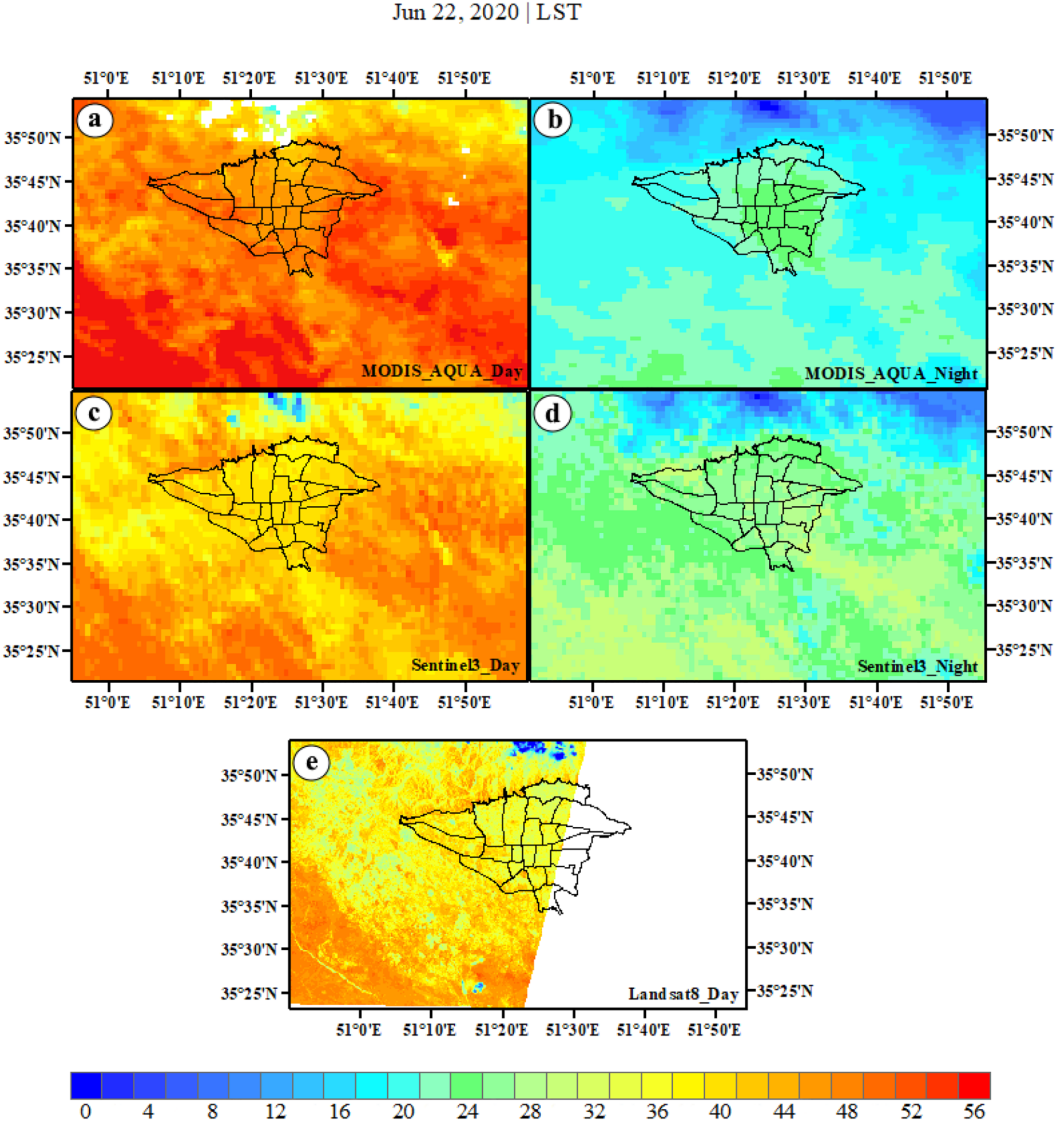
The images of LST captured by MODIS Aqua (**a**,**b**), Sentinel-3 (**c**,**d**), and Landsat 8 (**e**) satellites during summer are presented in both day and night. The data were prepared using Earth data. Cite: AppEEARS Team. Application for Extracting and Exploring Analysis Ready Samples (AppEEARS). Ver. X.X. NASA EOSDIS Land Processes Distributed Active Archive Center (LP DAAC), USGS/Earth Resources Observation and Science (EROS) Center, Sioux Falls, South Dakota, USA. https://appeears.earthdatacloud.nasa.gov/ (2020). Copernicus. ESA. Cite: E.U. Copernicus Marine Service Information (CMEMS). Marine Data Store (MDS). https://doi.org/10.48670 (2020). Earth explorer. USGS. Cite: U.S. Geological Survey. https://earthexplorer.usgs.gov/ (2020). The Sentinel-3 were produced in SNAP. Zuhlke et al.^111^, version number: 8, URL link: https://sentinels.copernicus.eu (2015). The maps were generated in ArcGIS. ESRI ArcGIS Desktop. Release 10. Redlands, CA: Environmental Systems Research Institute. Version number: ArcGIS 10.8. URL link: https://www.esri.com (2020).

Generally, both day and night images show that the diurnal pattern of LST is somewhat similar across all three satellites. However, MODIS Aqua provides a better reflection of nighttime LST compared to Sentinel-3. Additionally, during the day, the highest LST is observed in suburbs while at night it is observed in central urban areas (as shown in Fig. 2).

### The spatial investigation of UHI in three-satellite sensors

**Figure 3:**
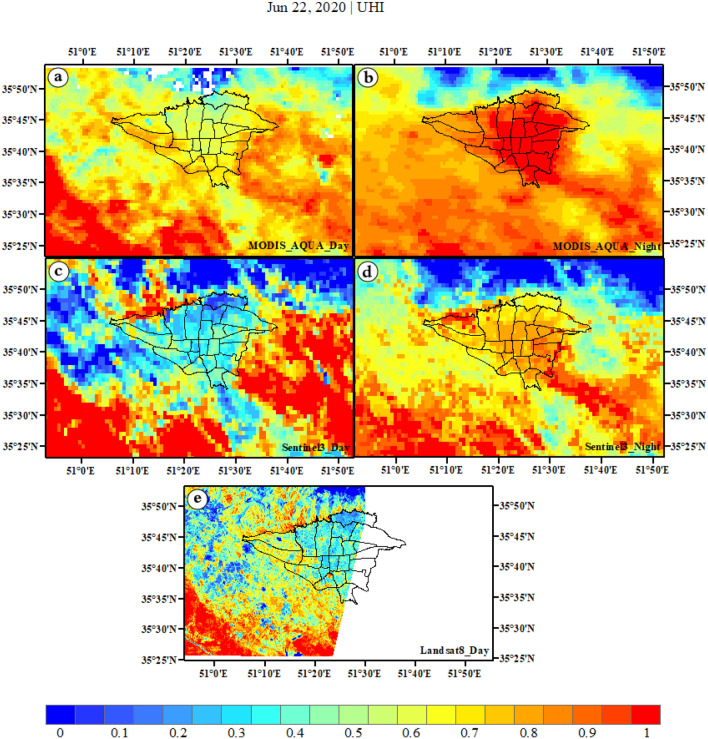
The images of UHI captured by MODIS Aqua (**a**,**b**), Sentinel-3 (**c**,**d**), and Landsat 8 (**e**) satellites during summer are presented in both day and night. The data were prepared using Earth data. Cite: AppEEARS Team. Application for Extracting and Exploring Analysis Ready Samples (AppEEARS). Ver. X.X. NASA EOSDIS Land Processes Distributed Active Archive Center (LP DAAC), USGS/Earth Resources Observation and Science (EROS) Center, Sioux Falls, South Dakota, USA. https://appeears.earthdatacloud.nasa.gov/ (2020). Copernicus. ESA. Cite: E.U. Copernicus Marine Service Information (CMEMS). Marine Data Store (MDS). https://doi.org/10.48670 (2020). Earth explorer. USGS. Cite: U.S. Geological Survey. https://earthexplorer.usgs.gov/ (2020). The Sentinel-3 were produced in SNAP. Zuhlke et al.^111^, version number: 8, URL link: https://sentinels.copernicus.eu (2015). The maps were generated in ArcGIS. ESRI ArcGIS Desktop. Release 10. Redlands, CA: Environmental Systems Research Institute. Version number: ArcGIS 10.8. URL link: https://www.esri.com (2020).

The UHI survey of Tehran is presented in Fig. 3, which includes daytime and nighttime images from MODIS Aqua, Sentinel-3, and Landsat 8 satellites. The results show that the lowest values are observed in the northern and western suburbs of Tehran during the day, followed by urban areas across all three satellites. However, these values gradually increase in the eastern, southeastern, southern, and southwestern suburbs. It is worth noting that in MODIS Aqua, sentinel-3, and Landsat 8 images, the values in urban areas are much lower than those in suburbs (a, c, e). In contrast to daytime images, nighttime images reveal a starkly different scenario where changes in UHI are much more noticeable. Urban areas with no UHI during the day have the highest values of UHI at night. Interestingly, compared to Sentinel-3, MODIS Aqua shows intense and robust UHI in urban areas at night, particularly in central areas (downtown Tehran) and some suburbs (southeast, south, southwest, and west). In MODIS Aqua and Sentinel-3 images during nighttime, UHI spreads significantly in urban and some suburban areas making it challenging to distinguish between urban and suburban borders. Unlike Sentinel-3, MODIS Aqua shows well-formed UHI in urban areas although its high values continue to some suburbs at night (b, d).

In general, the daily pattern is similar across all three satellites, although the nighttime pattern differs between them. Specifically, MODIS Aqua shows a better nighttime UHI than Sentinel-3. The highest daily values are observed in the suburbs, while the lowest are found in the city. However, during nighttime, UHI spreads significantly in Tehran's urban areas, particularly in central urban and some small suburban areas. Thus, this phenomenon is not limited to the 22 urban districts but has also spread beyond the city borders. As a result, the highest UHI values are in the city center and in the southeast, south, southwest, and west rural areas. Notably, there are high values in the lower suburbs (southeast, south, southwest) during both day and night across all satellites. Conversely, due to the presence of mountains, northern suburbs have the lowest values during both day and night.

### The LST changes from suburban to urban areas in three-satellite sensors

**Figure 4:**
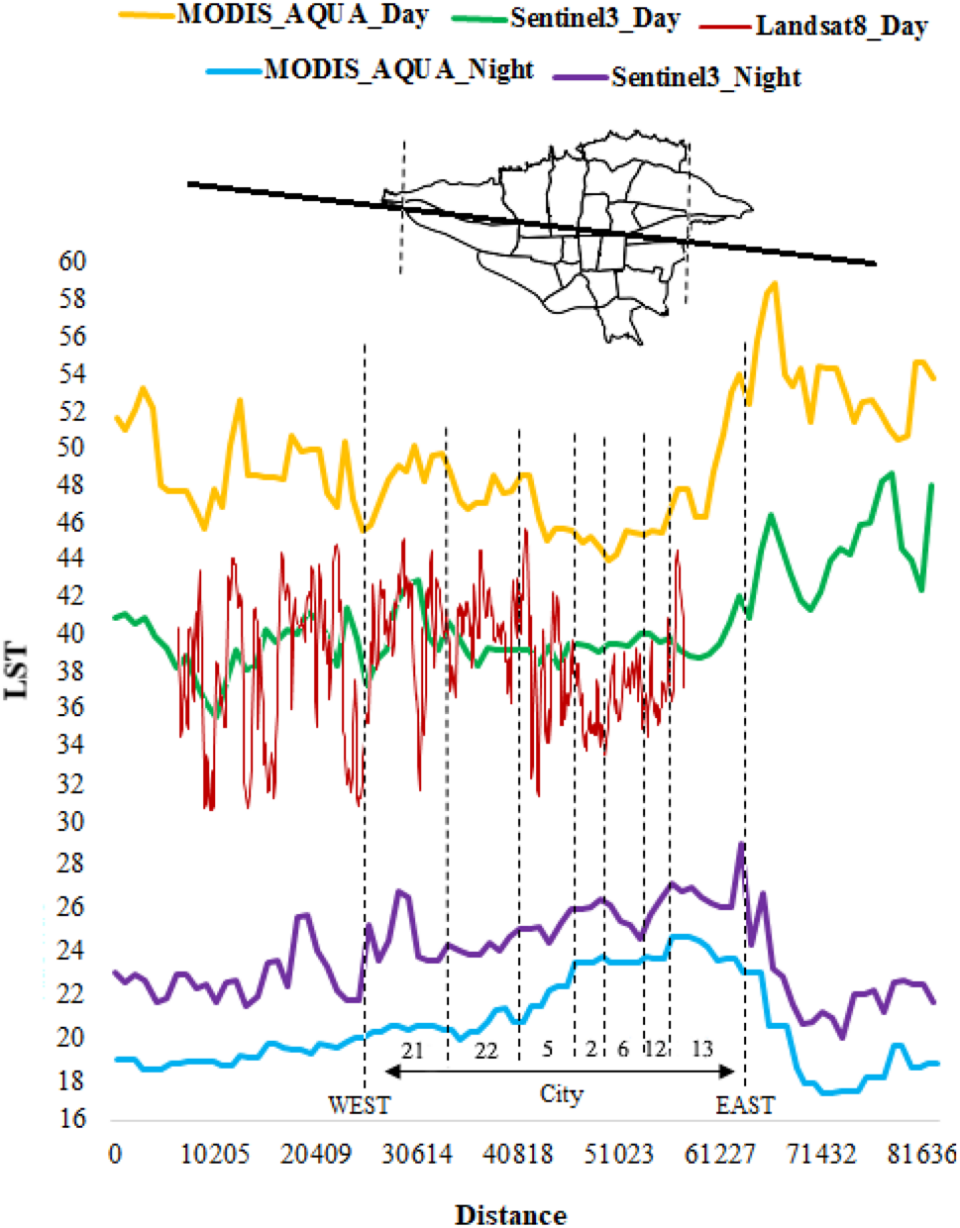
The LST variation from suburban to urban areas during day and night. Each number on the figure represents a specific district. The data were prepared using Earth data. Cite: AppEEARS Team. Application for Extracting and Exploring Analysis Ready Samples (AppEEARS). Ver. X.X. NASA EOSDIS Land Processes Distributed Active Archive Center (LP DAAC), USGS/Earth Resources Observation and Science (EROS) Center, Sioux Falls, South Dakota, USA. https://appeears.earthdatacloud.nasa.gov/ (2020). Copernicus. ESA. Cite: E.U. Copernicus Marine Service Information (CMEMS). Marine Data Store (MDS). https://doi.org/10.48670 (2020). Earth explorer. USGS. Cite: U.S. Geological Survey. https://earthexplorer.usgs.gov/ (2020). The maps first were generated in QGIS. QGIS.org. QGIS Geographic Information System. Open Source Geospatial Foundation Project. Version number: QGIS-OSGeo4W-3.16.4-1.URL link: http://qgis.org (2021) and then in paint.net. Version number: paint.net 4.3.11. URL link: https://www.getpaint.net (2022).

The latitudinal profile from west to east is illustrated in Fig. 4, which analyzes the difference in LST between day and night from rural to urban areas using multiple satellites. The distance between the western starting point and eastern ending point is 25,000 and 63,000, respectively the daily satellite images indicate a decline in temperature values from the rural areas towards the western part of the city. This temperature decrease persists until the start of the 21st district in the west, with temperatures reaching their lowest point in the center and east. Essentially, there is a more substantial rise in temperature towards the west than towards the center and east of Tehran. The study reveals that the eastern rural areas of Tehran have higher temperatures compared to the western rural areas. However, as one moves towards the center and east of the city, there is a decrease in temperature. The nighttime conditions are markedly different from the daytime images. The temperature axis indicates an increase in LST intensity from the western suburbs to the beginning of the city's west. As one moves towards the center and east of the city, LST intensity reaches its peak and then sharply decreases in the eastern suburban areas. In these images, the LST values exhibit a more pronounced temperature decline in the eastern suburban regions compared to the western suburban regions. It is worth noting that LST graphs indicate that areas that experience a decline in temperature during the day witness a rise at night, with higher values during the day corresponding to lower values at night and vice versa. In comparison to daily images, LST values are considerably higher at night in western rural areas and central urban areas. In an urban center, there appears to be less energy loss due to latent heat evaporation from impervious surfaces compared to rural areas. This results in a higher rate of heat storage on urban surfaces.

### The relationship between elevation geographical factor and Tehran’s UHI in three-satellite sensors

**Figure 5:**
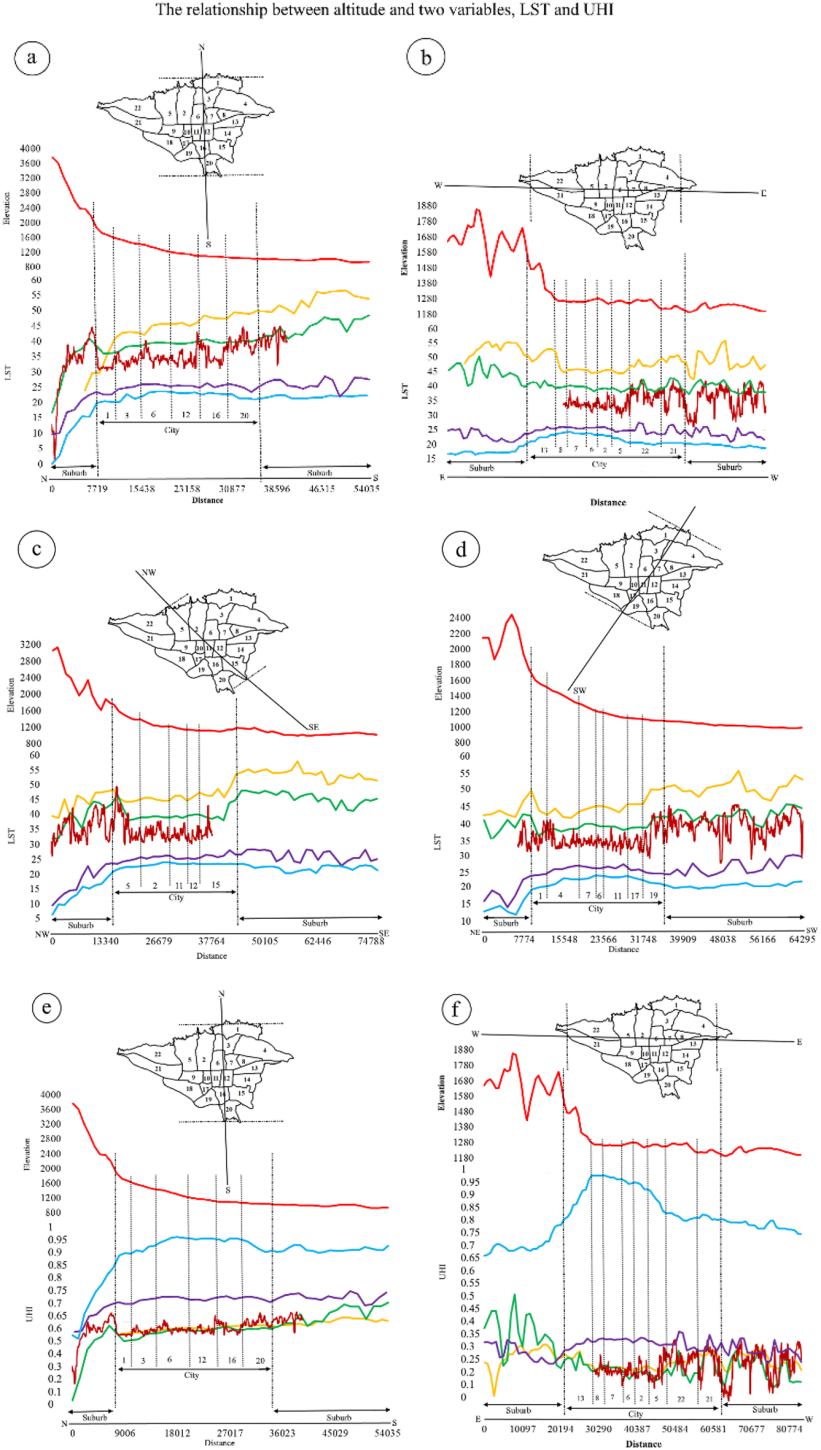

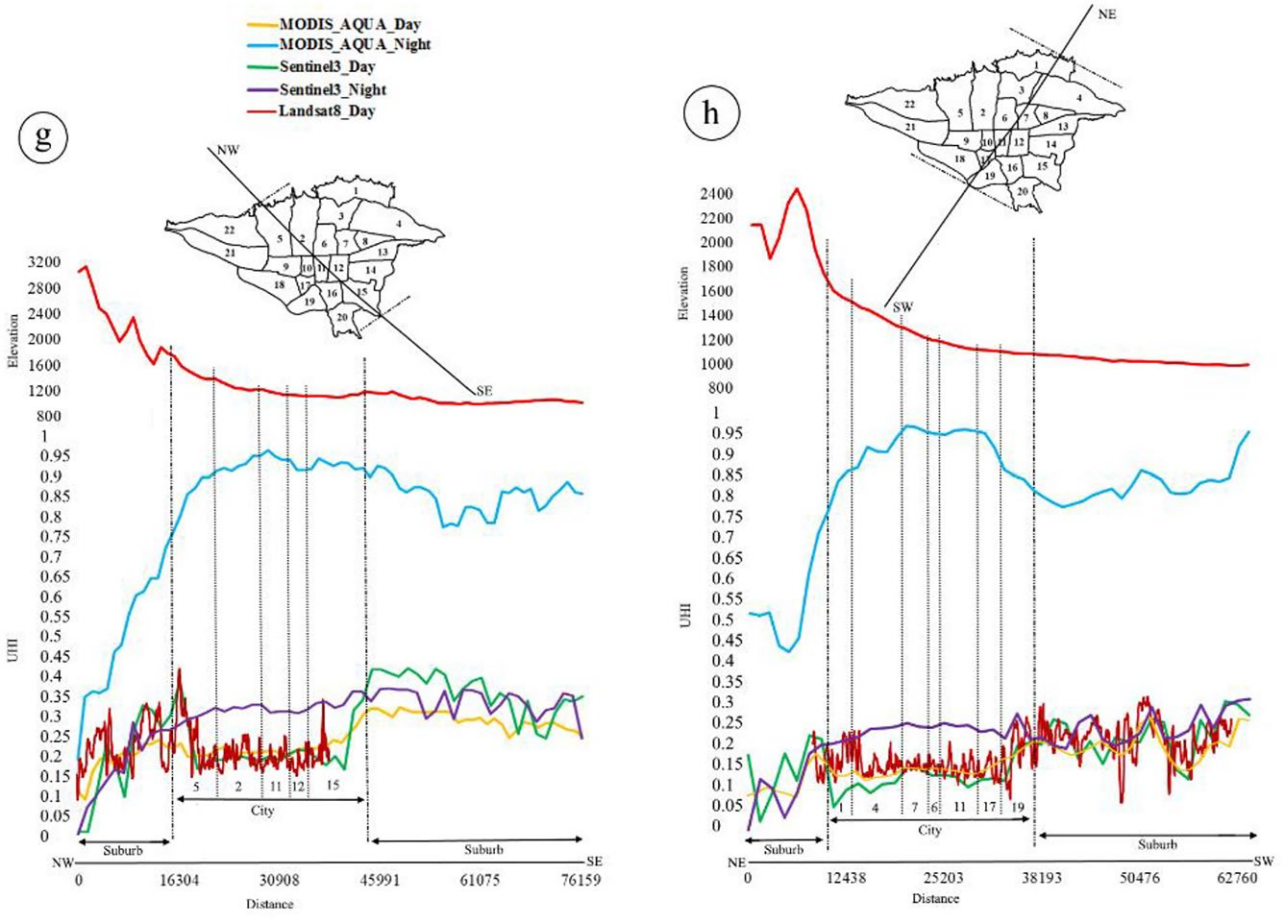
The correlation profile between height and LST (**a**–**d**), and between height and UHI (**e**–**h**) in urban and suburban areas (numbers in the figure represent urban areas). The data were prepared using Earth data. Cite: AppEEARS Team. Application for Extracting and Exploring Analysis Ready Samples (AppEEARS). Ver. X.X. NASA EOSDIS Land Processes Distributed Active Archive Center (LP DAAC), USGS/Earth Resources Observation and Science (EROS) Center, Sioux Falls, South Dakota, USA. https://appeears.earthdatacloud.nasa.gov/ (2020). Copernicus. ESA. Cite: E.U. Copernicus Marine Service Information (CMEMS). Marine Data Store (MDS). https://doi.org/10.48670 (2020). Earth explorer. USGS. Cite: U.S. Geological Survey. https://earthexplorer.usgs.gov/ (2020). The maps first were generated in QGIS. QGIS.org. QGIS Geographic Information System. Open Source Geospatial Foundation Project. Version number: QGIS-OSGeo4W-3.16.4-1.URL link: http://qgis.org (2021) and then in paint.net. Version number: paint.net 4.3.11. URL link: https://www.getpaint.net (2022).

This study aims to explore the correlation between height and two variables, namely LST and UHI. Figure 5 presents an elevation profile of Tehran, where the vertical axis represents height, LST, and UHI, while the horizontal axis represents distance. The objective is to analyze how elevation impacts LST and UHI. Tehran is situated amidst rugged terrain, with the northern and eastern parts of the city being particularly hilly. The highest altitude in the city is found in the north, at 1924 m, while the southern part of the city sits at a lower altitude of 1039 m. The height profile from northeast to southwest ranges from 1079 to 1780 m, while the northwest-southeast profile ranges from 1456 to 1177 m. Heights gradually decrease from east to west, ranging from 1369 to 1190 m. In the center of the city, there is a gentle slope with short elevations that takes a downward slope again in the west. As such, there is a continuous downward trend in height from north to south, northeast to southwest, and northwest to southeast. However, there is an upward trend from west to east accompanied by low elevations in the center. The altitude distribution of Tehran's regions reveals that Region 20 has the lowest altitude at 1100 m while region 1 has the highest altitude at 1900 m. Regions 15,16,18,19 and 20 have lower altitudes compared to Regions 1 and 2.

Based on the gradual decrease in altitude from north to south in the daily satellite images, the lowest temperature values are observed in the northern suburbs and urban areas (District 1), which gradually increase towards the south (zone 20) and especially in the southern suburbs. In other words, there is an increasing temperature gradient in all three satellites due to the decrease in altitude, resulting in higher temperature values as one moves from north to south through urban areas and into the southern suburbs. In MODIS Aqua and Sentinel-3 nighttime images, the LST curve has a consistent slope across different urban areas, continuing into the southern suburbs. As a result, the highest temperatures occur in central and southern regions of Tehran, while the lowest temperatures occur in northern suburbs due to their lower altitude (a).

In the east–west curve, the height gradually decreases from the eastern starting point to District 7, followed by mild fluctuations until District 5, and then a further decrease towards the west and western suburbs. Daily satellite images in the east–west direction reveal a rising temperature trend in both the eastern and western suburbs compared to urban areas. However, these conditions differ during night-time images, where temperatures increase significantly in urban areas, particularly in Districts 13 and 5. Conversely, suburban areas experience a decrease in temperature, which is more evident in MODIS compared to Sentinel-3 (b). The study reveals that there is a consistent decrease in altitude from the northwest to the southeast in daily images, resulting in the lowest temperature being recorded in the city center as compared to the suburbs. Conversely, the southeast suburbs experience higher temperatures than the northwest. In night, however, the lowest temperature was observed in the northwest suburbs while the highest was recorded in urban areas up to the southeast suburbs. This can be attributed to the fact that the center and south of the city and southeast suburbs have lower altitudes, resulting in higher temperatures (c).

As altitude decreases from the northeast to the southwest, the lowest daytime temperature values are observed in the northeast suburbs. A downward trend is observed up to zone 4, followed by an increasing trend with a sharp climb towards the southwest suburbs and urban areas. During nighttime, the highest rising temperature values are observed in urban areas (zones 1 to 19) and southwest suburbs compared to those in the northeast suburbs (zone d).

It is important to note that altitude plays a significant role in temperature fluctuations during both day and night in urban and suburban areas. Specifically, as altitude decreases, temperatures tend to increase.

In Tehran, there is a consistent decrease in height from north to south in the suburbs, resulting in increasing temperatures during the day. Conversely, at night, the UHI has higher values and a stronger upward trend over urban areas compared to daytime. As a result, the highest values are observed in Regions 1 to 20, followed by the southern suburbs, while the lowest values are found in the northern suburbs (e).

The highest values are observed in the eastern suburbs, followed by a decreasing trend towards the center of the city (District 5). However, there is a sharp rise towards the west and western suburbs. It is important to note that UHI conditions at night are opposite to those during the day, resulting in the eastern suburbs experiencing the lowest values. The trend from the east of the city to the center is slow and gradually decreases in intensity in areas 5 and 22. The trend then increases again with short dips and rises as it moves towards the west (f). During the day, suburban areas exhibit higher temperature values compared to the city. Specifically, in the northwest (District 5), there is a decrease in temperature in Region 15, followed by a strong upward trend towards the southeast. Conversely, at night, the UHI effect causes a significant increase in temperature in the city center, reaching its peak value at this location before fluctuating slightly towards the southeast of the city (g). In a northeast to southwest direction, there is an initial decreasing trend of values from Region 1 to Region 11. However, from Regions 17 and 19, there is a significant upward slope that continues towards the southwest suburbs. In the night, the UHI exhibits an upward trend from Regions 1 to 19. Generally, the highest UHI is observed in the center of the city compared to the suburbs (h).

Overall, It is observed that urban areas have lower temperatures than suburbs during the day across all satellites. However, at night, the temperatures rise over the city and some suburbs. Additionally, LST values increase during both day and night in all suburbs located in the south, southeast, and southwest.

The UHI values in urban areas have significantly decreased compared to suburbs during the day, as shown by the curves of UHI values with height in different directions across all three satellites. However, it is interesting to note that UHI values increase not only over the city but also over the suburbs in the south, southeast, southwest, and west at night. UHI values increase in the city compared to the suburbs in the north, northeast, and northwest. Additionally, UHI values extend beyond the city borders into these suburbs of the south, southeast and southwest.

MODIS Aqua shows a distinct difference in UHI compared to Sentinel-3 at night. However, both satellites show a similar general pattern during both day and night. The representation of LST and UHI is better when height decreases at night. In addition to natural elevation factor, vegetation, radiation, and cloud cover, the growth of suburbs in the south, southeast, southwest, and west has led to a reduction in distance between city and suburbs resulting in an expansion of UHI beyond city borders.

Based on a comparison of various satellites, it has been determined that MODIS Aqua is capable of accurately detecting UHI in Tehran. Therefore, our attention will be solely focused on the results obtained from MODIS Aqua.

### Moran's Global Index in MODIS Aqua

Table 7 displays the spatial autocorrelation values for Tehran's LST in various seasons, using Moran's global method. The results of the global Moran's spatial autocorrelation demonstrate that if the output statistic value is near a positive number (+ 1), then the data exhibit spatial autocorrelation and a cluster pattern. Conversely, if the index value is close to a negative number (− 1), then the data are dispersed. In simpler terms, when Moran's index value exceeds zero, the data display some form of spatial clustering, whereas when it falls below zero, they exhibit a scattered pattern. Therefore, this table provides valuable insights into Tehran's LST patterns across different seasons.

**Table 7:** The output of Moran's statistics for the LST in Tehran.

Seasons	Time	Moran's	Moran's index	Z-score	p-value	Variance
Spring	Day	0.932616	− 0.000052	202.055588	0.000000	0.000021
	Night	0.981734	− 0.000052	186.660275	0.000000	0.000028
Summer	Day	0.958360	− 0.000053	184.200961	0.000000	0.000027
	Night	0.996596	− 0.000053	208.578658	0.000000	0.000023
Autumn	Day	0.947016	− 0.000054	183.555507	0.000000	0.000027
	Night	0.986665	− 0.000052	212.266448	0.000000	0.000022
Winter	Day	0.809983	− 0.000053	130.490789	0.000000	0.000039
	Night	0.814098	− 0.000054	172.212411	0.000000	0.000022
Annual	Day	0.923576	− 0.000053	166.882700	0.000000	0.000031
	Night	1.006099	− 0.000053	176.833849	0.000000	0.000032

In the global Moran index, the null hypothesis assumes that there is no spatial clustering among the values of the element related to desired geographical features. However, if the p-value is very small and the calculated z-value (its absolute value) is immense, this null hypothesis can be rejected. The numerical output of Moran's index for different seasons indicates that summer and autumn have the highest values while spring and winter have the lowest values, both during day and night. In terms of seasonal and annual analysis, it has been observed that index values are higher at night than during the day. It is noteworthy that the value of Moran's index remains positive during both day and night in all seasons as well as throughout the year, tending towards + 1. Furthermore, a small p-value and large absolute value of the calculated z-value indicate spatial autocorrelation in the data with a clustered distribution pattern. If the temperature of LST is distributed uniformly in space, then the Moran's index for spring (day/night) and autumn (night) should be − 0.000052, while for summer (day/night), winter (day), and Annual (day/night), it should be − 0.000053. For autumn (day) and winter (night), it should be − 0.000054. The results of the global Moran's index suggest that the null hypothesis, which states that LST spatial correlation is not observed in Tehran, must be rejected. The analysis reveals that LST data in Tehran exhibit a spatial structure that is distributed in clusters.

The p-value is a statistical indicator that determines whether the results obtained are significant or due to chance. In the Moran statistic, a p-value of zero and less than 0.05 indicates that the results obtained from LST in Tehran are significant, and there is a low probability that they occurred randomly, leading to the rejection of the null hypothesis. LST in Tehran has a clustered distribution.

### The Getis-ord Gi* index in MODIS Aqua

**Figure 6:**
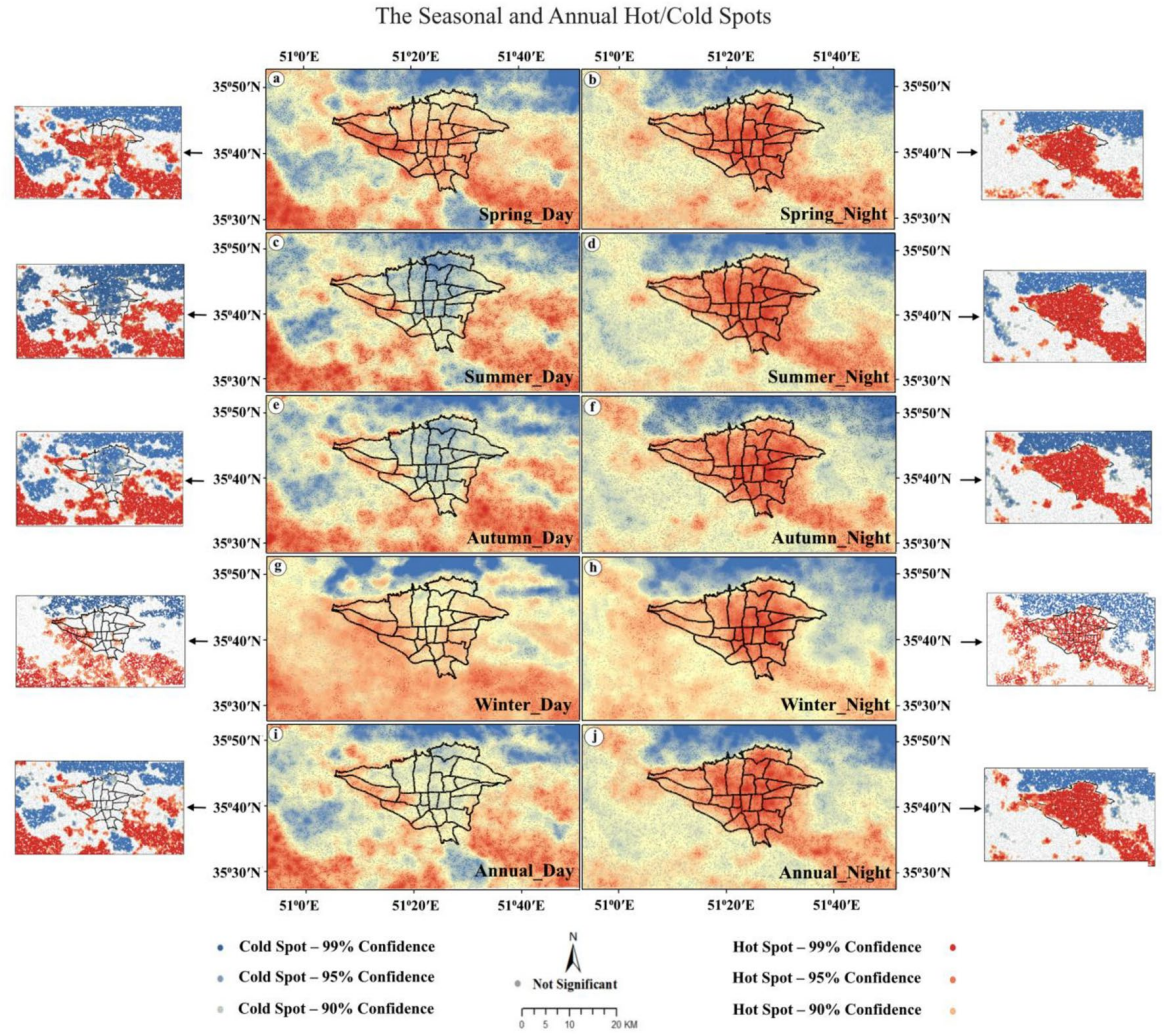
The seasonal/annual cold-spot (**a**,**c**,**e**,**g**,**i**) and hot-spot (**b**,**d**,**f**,**h**,**j**) within urban and rural using the Getis-Ord Gi* statistic. The data were prepared using GEE. Cite: Gorelick et al.^112^. The maps were generated in ArcGIS. ESRI ArcGIS Desktop. Release 10. Redlands, CA: Environmental Systems Research Institute. Version number: ArcGIS 10.8. URL link: https://www.esri.com (2020), and then in paint.net. Version number: paint.net 4.3.11. URL link: https://www.getpaint.net (2022).

The study utilized the Getis-Ord Gi* index to identify areas with high and low-value clusters. The results of the Getis-Ord Gi* statistics are presented in seasonal and annual maps in Fig. 6.

The hot-spot analysis conducted in this study was addressed to detect urban areas with nighttime LST values showing spatially widespread LST values and thus creating positive or negative structural LST anomalies. Getis-Ord Gi* statistic is the method used to identify statistically significant spatial clusters of high (hot-spot) and low LST values (cold-spot) at local scale.

The Getis-Ord Gi* statistic calculates a z-score for each point in the data, indicating whether high or low data values are clustered in specific regions. A positive, large, and significant z-score indicates intense clustering of hot spots with high values, while a small, negative, and significant z-score indicates extreme clustering of low values forming cold spots. In seasonal maps, red spots indicate hot spots with larger values than z, while blue spots indicate cold spots formed when z decreases and becomes smaller. The UHI is classified as hot or cold based on the 90%, 95%, and 99% confidence level and lack of significance level. Tehran is classified within the confidence level of 90 to 99%.

At night, over Tehran, all of the z scores are larger than 2.58 (Hot-spot 99). Features in this area surrounded by features with high values. Hence, the area shows the high-high cluster, which means viral hepatitis has a high incidence in this area. And so, in this area, people should take preventive measures more seriously. The southeast, south, southwest, and northwest suburbs are between 1.65 to 1.96 (95% to 90%) which show hot-spot in these areas. Z scores in gray areas are closer to 0 without statistical significance. However, during day, z scores in urban areas show cold-spot.

During spring and winter, hot spots are present in most urban areas, as well as the suburbs located in the southeast, south, and southwest. Conversely, during summer and autumn, cold spots can be observed in the city and suburbs located in the north and west, while hot spots are present in the southeast, south, and southwest regions. Cold spots are only seen during these seasons in some suburbs located in the north, east, west, and south. Despite these daily variations between seasons, UHI is consistently formed over the city at night throughout all seasons. This effect is particularly pronounced in the suburbs located in the southeast, south, southwest, and west where more hot spots are observed. Due to this annual distribution, cold spots form during the day within the city and most of its suburbs while hot spots form at night within the city itself as well as its suburbs located in the southeast, south, southwest, and west.

Overall, although there are differences in daily patterns between seasons (spring/winter versus summer/autumn), a similar night pattern is observed throughout all seasons. on an annual average, there is UCI present during the daytime while UHI dominates at night. Additionally, it is noteworthy that significant hot spots exist both day and night within Tehran's southeast, south, southwest, and west suburbs.

### The nighttime temperature difference according to hot and cold spots in MODIS Aqua

**Figure 7:**
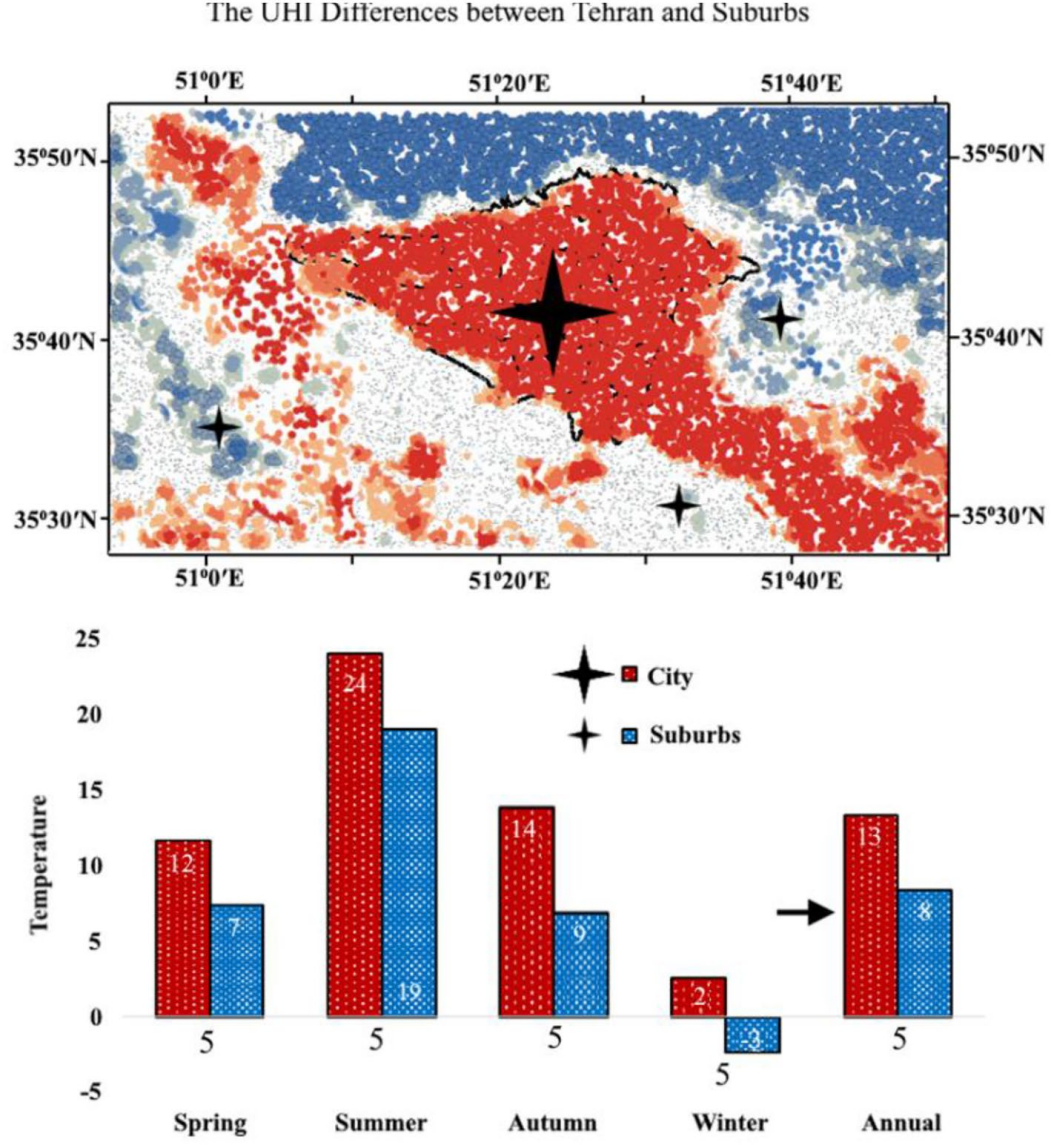
The seasonal and annual differences in UHI using hot (City) and cold (Suburbs) spots. The data were prepared using Earth data. Cite: AppEEARS Team. Application for Extracting and Exploring Analysis Ready Samples (AppEEARS). Ver. X.X. NASA EOSDIS Land Processes Distributed Active Archive Center (LP DAAC), USGS/Earth Resources Observation and Science (EROS) Center, Sioux Falls, South Dakota, USA. https://appeears.earthdatacloud.nasa.gov/ (2020). The maps first were generated in QGIS. QGIS.org. QGIS Geographic Information System. Open Source Geospatial Foundation Project. Version number: QGIS-OSGeo4W-3.16.4-1.URL link: http://qgis.org (2021) and then in paint.net. Version number: paint.net 4.3.11. URL link: https://www.getpaint.net (2022).

This section aimed to investigate the variations in UHI between hot and cold areas on a seasonal and annual basis (Fig. 7). The MODIS Aqua satellite was utilized to identify the hot and cold spots in downtown Tehran and three surrounding points, namely east, south, and west. A bar graph was created to calculate the temperature difference between the urban and rural areas. The results indicated that both the city (24 °C) and suburbs (19 °C) had their highest temperature values during summer, which differed significantly from other seasons. Spring (12 °C and 7 °C) and autumn (14 °C and 9 °C) had slightly lower temperatures, while winter (2 °C and − 3 °C) had the lowest values for both areas. The annual difference between the city and suburbs was 13 and 8, respectively. The temperature difference between them was 5 °C in all seasons and year. Interestingly, there was a decrease in temperature difference between Tehran and its suburbs due to suburban growth. The study revealed significant seasonal and annual differences in UHI between hot and cold areas. During summer, UHI were higher in hot areas than cold ones; however, during winter, UHI were lower in hot areas than cold ones.

### The temporal distribution of LST in MODIS Aqua

**Figure 8:**
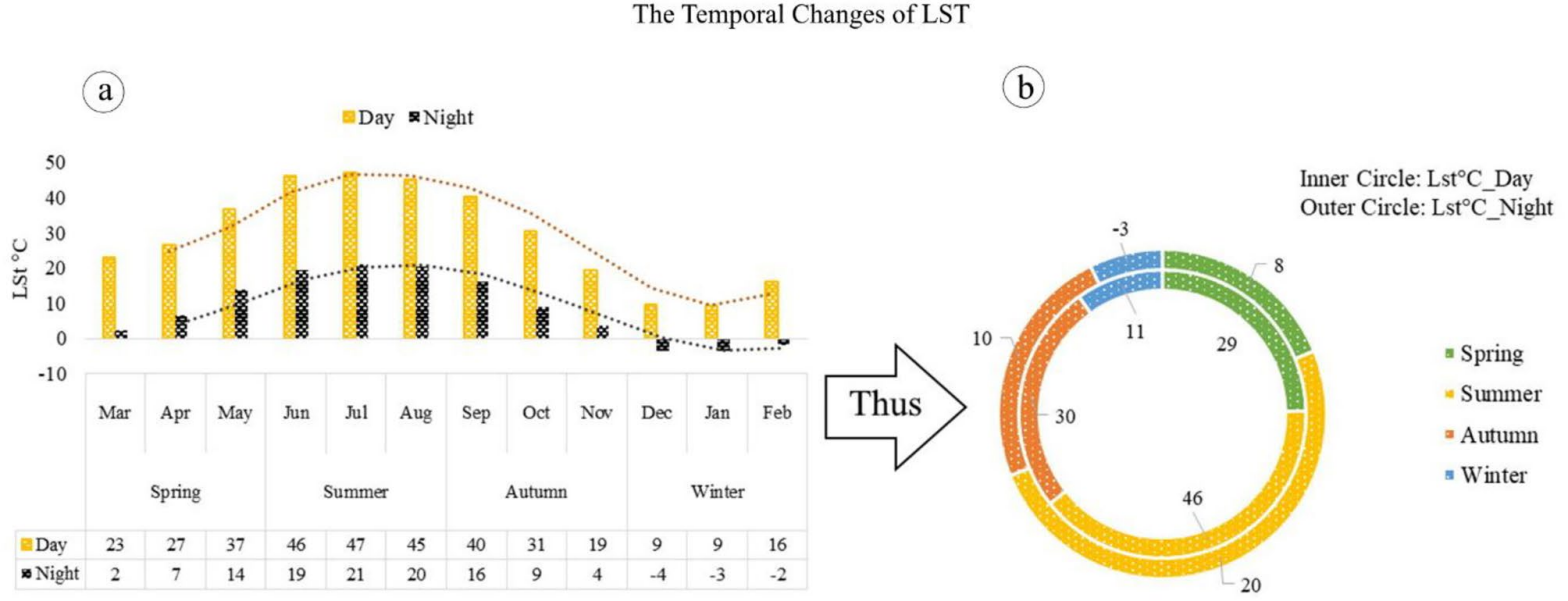
The monthly (**a**) and seasonal (**b**) average of maximum (1:30 p.m. local time) and minimum (1:30 a.m. local time) LST in Tehran. The data were prepared using GEE. Cite: Gorelick et al.^112^. The maps were generated in Microsoft Excel. Microsoft Corporation. Microsoft Excel, Version number: Excel 2019.URL link: https://office.microsoft.com/excel (2018), and then in paint.net. Version number: paint.net 4.3.11. URL link: https://www.getpaint.net (2022).

Figure 8 displays an analysis of the average characteristics of LST during different seasons, both during the day and at night. The data show that April and March have the lowest temperatures, with values of 27 and 23 °C during the day and 7 and 2 °C at night. In contrast, May has the highest temperature, with values of 37 °C during the day and 14 °C at night. This leads to a positive trend in June and July, with numerical values of 46 and 47 °C during the day and 19 and 21 °C at night. However, there is a decrease in temperature in August, September, October, and November. During these months, daily temperature values are 45, 40, 31, and 19 °C respectively while night temperatures are around 20, 16, 9, and 4 °C. The autumn season has an upward trend compared to spring but still has lower temperatures than summer. During winter, there is a significant decrease in temperature with December and January experiencing the lowest levels (9 °C during the day). In these months, nighttime temperatures reach − 4 and − 3 °C while February experiences its lowest temperature with values of 16 and 2 °C for day and night respectively (a). Based on this, it is more evident that there is an increase in LST during the summer, both during the day (46 °C) and night (20 °C), compared to other seasons. Autumn has a slightly higher average temperature (30 and 10 °C) than spring (29 and 8 °C). The winter has the lowest average temperature, with daytime temperatures averaging at 11 °C and nighttime temperatures averaging at − 3 °C (b).

### The temporal distribution of NDVI in MODIS Aqua

**Figure 9:**
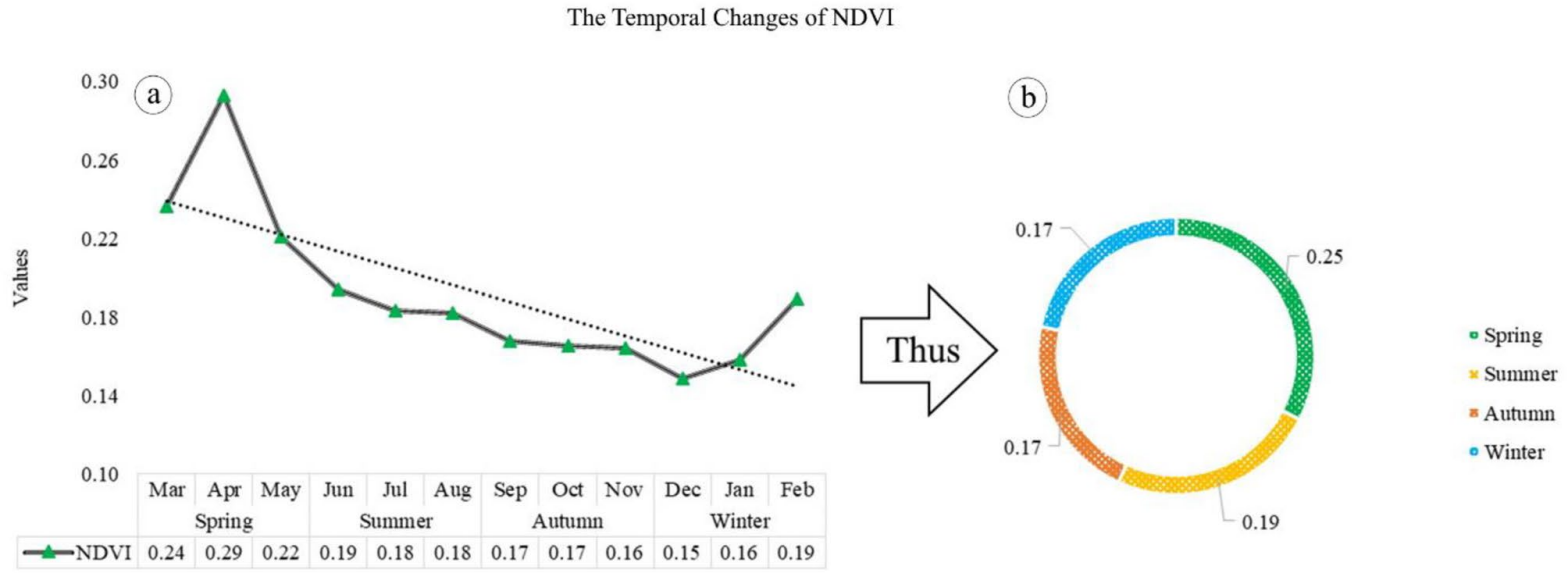
The monthly (**a**) and seasonal (**b**) distribution of NDVI index in Tehran. The data were prepared using GEE. Cite: Gorelick et al.^111^. The maps were generated in Microsoft Excel. Microsoft Corporation. Microsoft Excel, Version number: Excel 2019.URL link: https://office.microsoft.com/excel (2018), and then in paint.net. Version number: paint.net 4.3.11. URL link: https://www.getpaint.net (2022).

Figure 9 displays the vegetation index for Tehran during different seasons, revealing that the highest NDVI occurs in April with a value of 0.29 compared to March and May. This trend gradually declines until other months, with NDVI reaching 0.19 in June and 0.18 in July and August. From September to October, NDVI remains at 0.17, and this decreasing trend continues until November (0.16) and December (0.15), with an increase to 0.16 and 0.19 in January and February, respectively (a). Therefore, the highest NDVI index according to seasonal division is observed in spring (0.25), while the other seasons have the lowest values. The decrease in NDVI during autumn and winter is the primary reason for reduced vegetation activity during the growing season (b).

In Table 8, the NDVI values were classified into three categories: no, weak, and strong vegetation cover. This classification was based on threshold values determined by analyzing the distribution of NDVI values. The results indicate that the majority of urban areas in Tehran have either no or weak vegetation cover. In general, based on the NDVI index classification, urban areas in Tehran experience a lack of vegetation from summer to winter, although there is some weak vegetation during spring.

**Table 8:** Urban vegetation classification using NDVI threshold values in Tehran.

Row	vegetation classification	Description	values	NDVI
1	No vegetation	Bair and Built-up areas, road network	− 1 to 0.199	Summer Autumn Winter
2	Weak vegetation	Bushes and meadows	0.2 to 0.5	Spring
3	Strong vegetation	temperate and tropical urban forest	0.51 to 1	–

### The spatial distribution of LST, UHI and NDVI in MODIS Aqua

**Figure 10:**
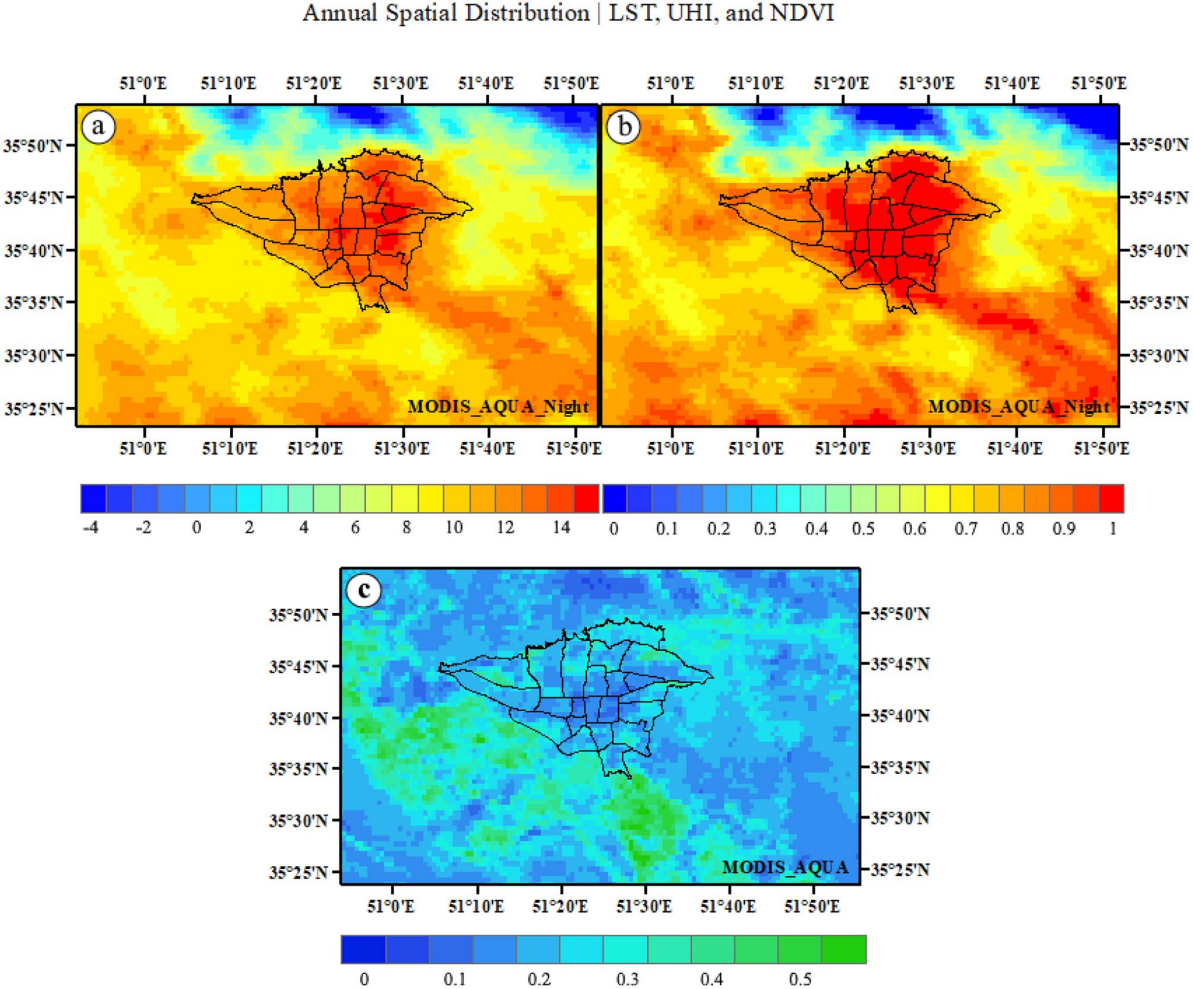
The spatial distribution of annual LST (**a**), UHI (**b**), and vegetation (**c**) in Tehran. The LST/UHI data were prepared using GEE. Cite: Gorelick et al.^112^. The NDVI data were prepared using Earth data. Cite: AppEEARS Team. Application for Extracting and Exploring Analysis Ready Samples (AppEEARS). Ver. X.X. NASA EOSDIS Land Processes Distributed Active Archive Center (LP DAAC), USGS/Earth Resources Observation and Science (EROS) Center, Sioux Falls, South Dakota, USA. https://appeears.earthdatacloud.nasa.gov/ (2020). The maps were generated in ArcGIS. ESRI ArcGIS Desktop. Release 10. Redlands, CA: Environmental Systems Research Institute. Version number: ArcGIS 10.8. URL link: https://www.esri.com. (2020).

This section analyzes the spatial distribution of LST, UHI, and NDVI in MODIS Aqua. According to Fig. 10, LST increases during the night in the downtown Tehran and southeastern, southern, southwestern, and western rural areas, while it decreases in the northern suburbs. There is a significant variation in LST across different land use types, with urban areas having higher LST values due to the UHI effect (a). Annual satellite images of UHI at night show that UHI increases in urban areas and extends beyond the city into some suburbs. The UHI effect also contributes to lower NDVI values in urban areas (b). The NDVI index also reveals areas with little or no vegetation in both urban and suburban areas. Most urban areas experience UHI throughout the year due to a lack of vegetation at night. However, if there is more green cover per capita in the city, then UHI effects are more significant during the day. Additionally, NDVI values are higher in suburban areas compared to urban areas because of greater vegetation cover in rural areas (c).

Overall, the spatial distribution indicates that the lack of NDVI index in urban areas leads to higher LST and UHI values. This study provides valuable insights into the spatial distribution of LST, UHI, and NDVI in MODIS Aqua.

## Discussion

**Figure 11:**
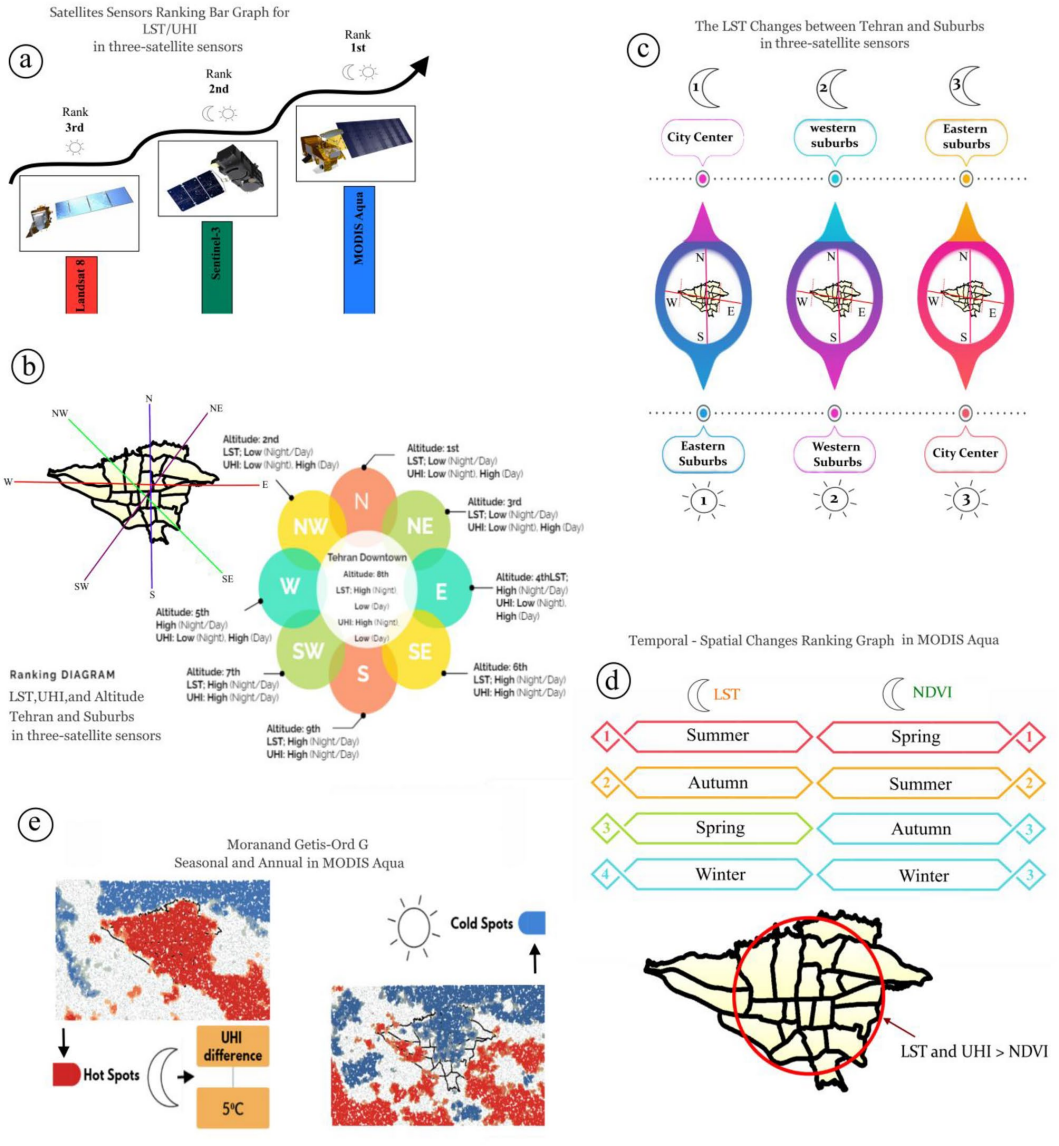
Visual Aid in Tehran.

Various satellite products, including MODIS Aqua, Sentinel-3, and Landsat 8, were compared and interpreted in order to select the best satellite for studying the SUHI in Tehran during day and night using GIS, GEE, QGIS, and SNAP software (Fig. 11). With the current advancements in thermal remote sensing observations, data retrieved from several sensors such as MODIS Aqua^83–85^, Landsat 8^86–89^, and Sentinel-3^90,91^ have been used to identify UHI. The available data with high spatial and temporal resolution have provided suitable opportunities to overcome the current limitations of ground-based weather data^34^. The use of Landsat's 100-m thermal data with a temporal resolution of 16 days has created limitations for this satellite due to weaknesses in detecting urban heat intensity without considering scale refinement^92^, sharpening techniques^93^, image fusion^94^, and disaggregation^95^ as effective methods to increase temporal resolution. Sentinel-3 contains thermal channels that enable the calculation of LST. For monitoring phenomena like LST and UHI in this satellite is crucial during the information source period because it can be detected most accurately at that time since objects have the highest heat during the day and emit the most thermal energy at night^96^. However, due to inappropriate imaging time of Sentinel-3 during dawn and high nocturnal longwave radiation in Tehran city, this satellite also lacks necessary efficiency. On the other hand, MODIS LST data with a spatial resolution of 1 km and a temporal resolution of 12 h has suitable capabilities for estimating urban heat^97^. Additionally, Aqua LST products were chosen instead of Terra for this study because Aqua's passing time is much closer to the actual occurrence of maximum and minimum temperatures observed and also closer to VIIRS's passing time at night^98^.

Among various platforms, given the significance of MODIS in receiving thermal radiations between the surface and atmosphere, a wide range of studies have utilized thermal bands from different products to retrieve surface temperature, assess climate, anomalies, and their trends^99–103^. In previous research, various satellites have been used to investigate UHI in Tehran^104,105^. However, none of them have addressed which satellite is more suitable and practical for studying SUHI in Tehran and accurately representing the regional climatic conditions. The value of this study lies in determining that among the investigated satellites, MODIS Aqua satellite with its appropriate imaging time (1:30 A.M.) is the best satellite for studying SUHI in Tehran. Furthermore, no research has been conducted to examine the UHI difference in Tehran along with its surrounding suburbs. Thus, since UHI has a greater impact during summer nights in Tehran, June 22nd, 2020 was chosen as a sample day among the three satellites to focus the analysis on summer night SUHI in Tehran (a).

Based on the characteristics of each region, Tehran is divided into 5 areas including north, south, west, east, and central, as well as UHI. Each of these regions has prominent geographical, industrial, and commercial positions. Tehran is located between the mountain valleys (southern slopes of Alborz mountains) and the desert (northern margin of Iran's central desert). It is surrounded by hills in the east and plains in the west. It has developed from the north up to an altitude of 2000 m and from the south up to an altitude of 1000 m. Over the past four decades, the LST has shown a significant increase during summer in Tehran^26^. Although the elevation decreases from north to south and from east to west, UHI and LST increase in these areas (b).

The temperature difference between the north and south of the city ranges from 6 to 10 °C, indicating that maximum UHI and LST are located in the city center (areas 7 to 14), with higher values in the south compared to the north. Additionally, the temperature difference between the city center and its outskirts shows that the central area and west have higher temperatures compared to the eastern. The maximum UHII in Tehran occurs during warm nights. Additionally, unlike the high altitudes of the northern parts of Tehran (District 1), the central parts of the city deal with UHI due to their lower height and dense old buildings throughout warm and cold months of the year (c)^106^.

The green cover of Tehran city has decreased over the past 30 years (1990–2020). In terms of spatial distribution, areas with old green spaces scattered throughout the city have the highest green space ratio. However, some areas (1, 4, 8, 13, 14, 15, and 20) have the lowest green space and highest LST/UHI values. 60% of the changes in Tehran's vegetation cover index are attributed to urban growth and population density increase^107^. built-up classes including compact high-rise, compact mid-rise, and heavy industrial areas tended to increase the surface temperature, while except for bare land, all other land cover types tended to decrease the surface temperature^108^.

The organization of buildings can significantly impact wind flow, solar energy distribution, and energy balance in urban areas, ultimately affecting the LST^109^. In Tehran, LST is primarily influenced by the maximum temperature of hard surfaces such as asphalt, stone, cement, and bare soil. These surfaces have a high heat capacity and contribute to higher temperatures in urban areas compared to rural areas. The arrangement of buildings in urban areas can create urban canyons that prevent radiation emissions from core urban areas. Seasonal and diurnal variations in LST may be attributed to monthly variations in solar radiation and cloud cover over Tehran. Furthermore, in terms of time, summer and autumn have the lowest NDVI values and highest LST values.

Therefore, the highest UHI is observed during the night in the five regions of Tehran and its southeastern, southern, southwestern, and western suburbs^110^. The SUHI of Tehran has a close relationship with spatial–temporal changes in vegetation cover, transportation, and industrial activities. In fact, the expansion of Tehran's SUHI towards the west, southwest, south, and southeast is due to a significant decrease in vegetation cover and expansion of industrial-workshop activities as well as customs and warehousing. The western suburbs with sand mines and industrial towns not only contribute to temperature increase but also act as wind tunnels transferring air flows into the city (d). The climate of Tehran has undergone significant changes due to extensive urbanization. Uncontrolled expansion of Tehran, population growth in the east and south, increased traffic in the north and east, increased consumption of fossil fuels in construction sectors as well as transportation and industry sectors, increased greenhouse gas emissions, and high-rise construction especially in urban corridors have led to the expansion of SUHI within the city. Hotspots are formed across all five regions of Tehran especially in its center and southeastern outskirts resulting in a temperature difference of 5° between the city center and its outskirts. The southeastern outskirts are an important source for supplying construction materials for Tehran province with sand mines and concrete factories. In fact, sand with high thermal conductivity contributes to temperature increase in these areas (e).

## Conclusion

To investigate the SUHI phenomenon in Tehran, high-resolution satellite data from MODIS Aqua, Sentinel-3, and Landsat 8 were utilized. The investigation of SUHI during summer was more extensive compared to other seasons in Tehran.

The diurnal pattern of LST and UHI is quite similar across all three satellites, both during the day and at night. However, MODIS Aqua provides a better representation of night-time LST/UHI compared to Sentinel-3. It has been observed that during the day, the highest LST is found in suburbs, while at night, it is observed in central urban areas. This indicates that SUHI is not limited to the 22 urban districts but has also spread beyond the city borders. The highest SUHI values are observed in downtown areas and lower suburbs (southeast, south, southwest, and west) during night across all satellites. Conversely, due to the presence of mountains, northern suburbs have the lowest values during both day and night.

The variation in LST between rural and urban areas is evident from the stark difference in nighttime conditions as compared to daytime images. In contrast to the daily images, the LST values are significantly higher at night in the western suburbs and central urban areas, particularly in the MODIS Aqua image.

There are variations in the differences between LST and UHI with altitude in different directions. During the day, LST/UHI values in urban areas have significantly decreased compared to suburbs. However, at night, LST/UHI values increase not only over the city but also over suburbs in the south, southeast, southwest, and west. Additionally, UHI values extend beyond the city borders into these suburbs of the south, southeast, southwest, and west at night. MODIS shows a distinct difference in UHI compared to Sentinel-3 at night. However, both satellites show a similar general pattern during both day and night. The representation of LST/UHI is better when height decreases at night. In addition to natural elevation factors, the growth of suburbs in the south, southeast, southwest, and west has led to a reduction in distance between cities and suburbs resulting in an expansion of SUHI beyond city borders.

After conducting comparative studies and analyses among the three satellites, it was discovered that MODIS Aqua is better at identifying SUHI in urban areas of Tehran and its suburbs than Sentinel-3. As a result, only MODIS Aqua day and night images were used for further investigations.

The output of Moran's index indicates that during both day and night, summer and autumn have the highest values. Additionally, index values are higher at night than during the day in seasonal and annual analysis. The positive numerical values in both seasonal and annual analyses reveal that LST data in Tehran exhibit a spatial structure that is distributed in clusters.

The Getis-Ord G statistic was used to analyze the daily patterns in Tehran, with a focus on the differences between seasons. It was found that while there were variations in daily patterns between spring/winter and summer/autumn, a similar night pattern was observed across all seasons. On an annual average, UCI was present during the daytime, while UHI dominated at night. It is worth noting that significant hot spots were identified both during the day and at night in Tehran's southeast, south, and southwest suburbs. The primary factor influencing LST in Tehran was found to be the maximum temperature of hard surfaces with high heat capacity. This contributed to higher temperatures in urban areas compared to suburbs. Seasonal and diurnal variations in LST were attributed to monthly changes in solar radiation and cloud cover over Tehran. Overall, these results provide valuable insights into the patterns of UHI in Tehran and highlight the importance of considering both seasonal and diurnal variations.

The temperature difference between the city and suburbs indicating that both areas experienced their highest temperatures during summer, which was significantly different from other seasons. Additionally, the seasonal and annual nighttime SUHI difference between Tehran and its suburbs equated to 5 °C in MODIS Aqua night products. The research demonstrated significant seasonal and annual variations in UHI between hot and cold areas. During summer, UHI were higher in hot areas than cold ones; However, during winter, UHI were lower in hot areas than cold ones.

The monthly and seasonal LST diagrams indicate that temperatures are higher during the warm months. Specifically, summer, autumn, and spring experience higher temperatures than winter.

When examining distribution charts of NDVI, it becomes apparent that the months from June to February have lower temperatures compared to other months. The highest NDVI index is observed in spring, while the other seasons have lower values. In essence, NDVI reveals areas without vegetation in all seasons except for spring (which has weak coverage). Additionally, MODIS Aqua night images reveal that most urban areas in Tehran lack vegetation cover or have weak coverage. Urban areas in Tehran experience a lack of vegetation from summer to winter, although there is some weak vegetation during spring.

The spatial distribution of LST, UHI, and NDVI in MODIS-Aqua show that there is a significant variation in LST across different land use types, with urban areas having higher LST values due to the UHI effect. Furthermore, NDVI values are higher in suburban areas compared to urban areas due to greater vegetation cover in rural areas. The UHI effect also contributes to lower NDVI values in urban areas. Overall, the spatial distribution indicates that the lack of NDVI index in urban areas leads to higher LST and UHI values. This study provides valuable insights into the spatial distribution of LST, UHI, and NDVI in MODIS-Aqua.

It is recommended to use MODIS Aqua for studying LST and UHI at night in Tehran. SUHI is not limited to urban areas and has spread beyond the city borders, with the highest values found in downtown Tehran and its southeast, south, southwest, and west suburbs. LST/UHI values are higher during warm periods, particularly in summer. Decreased altitude and vegetation contribute to an increase in LST/UHI at night. Natural factors such as elevation and vegetation, along with the growth of suburbs in the south, southeast, southwest, and west have reduced the distance between city and suburbs resulting in an expansion of SUHI beyond city borders. The southeast, south, and southwest suburbs have high SUHI values during both day and night across all satellites. This study provides valuable insights into how SUHI changes during summer in Tehran that can inform urban planning strategies aimed at mitigating the negative effects of UHI on human health and well-being.

### Supplementary Information

Supplementary Information.

## Data Availability

The datasets generated and/or analyzed during the current study are available in the NASA's Earth Science Data Systems (ESDS) Program via NASA's Land Processes Distributed Active Archive Center (LP DAAC) website (https://www.earthdata.nasa.gov/). The following links provide direct access to the data used in the research: https://appeears.earthdatacloud.nasa.gov/; 10.5067/MODIS/MYD11A1.061. Also, Google (https://earthengine.google.com/), ESA (https://scihub.copernicus.eu/), USGS (https://earthexplorer.usgs.gov/).
