# Author Correction: Climatic comparison of surface urban heat island using satellite remote sensing in Tehran and suburbs

**DOI:** 10.1038/s41598-024-55490-y

**Published:** 2024-03-04

**Authors:** Motahhareh Zargari, Abbas Mofidi, Alireza Entezari, Mohammad Baaghideh

**Affiliations:** 1 Hakim Sabzevari University, Sabzevar, Iran; 2 Ferdowsi University of Mashhad, Mashhad, Iran

Correction to: *Scientific Reports* 10.1038/s41598-023-50757-2, published online 05 January 2024.

The original version of this Article contained an error in Reference 36, which was incorrectly given as:

Shumilo, L., Kussul, N., Shelestov, A., Korsunska, Y., Yailymov, B. Sentinel-3 urban heat ISLAND monitoring and analysis for KYIV based on vector data. In *10th International Conference on Dependable Systems, Services and Technologies (DESSERT)* 10.1109/dessert.2019.8770042 (2019).

The correct reference is listed below:

García, D.H. Analysis of Urban Heat Island and Heat Waves Using Sentinel-3 Images: a Study of Andalusian Cities in Spain. *Earth Syst Environ* **6**, 199–219. 10.1007/s41748-021-00268-9 (2022).

Furthermore, the work of Zuhlke *et al.* (2015) was incorrectly cited in the Figure Legends of Figure 2 and 3, respectively. As a result, this reference was omitted from the Reference list.

“Cite: Zuhlke, Marco, Norman Fomferra, Carsten Brockmann, Marco Peters, Luis Veci, Julien Malik and Peter Regner. SNAP (Sentinel Application Platform) and the ESA Sentinel 3 Toolbox. Version number: 8. URL link: https://sentinels.copernicus.eu (2015).”

now reads:

“Zuhlke *et al.*^111^, version number: 8, URL link: https://sentinels.copernicus.eu (2015).”

The original Article has been corrected.

