# Supplementary material for: Climatic comparison of surface urban heat island using satellite remote sensing in Tehran and suburbs
Source: Sci Rep. 2024 Jan 5;14:643. doi: 10.1038/s41598-023-50757-2 (PMC10770034; doi:10.1038/s41598-023-50757-2)
Supplement: Supplementary file 1 — Supplementary Information. [file 41598_2023_50757_MOESM1_ESM.docx]

**Appendices**

**Appendix A. Sentinel-3**

The SLSTR sensing instrument products use an algorithm based on radiative transfer theory to calculate radiation exchange between the surface and atmosphere. These algorithms implicitly take into account the effects of land surface emissivity through biome and fractional vegetation. The basic algorithm can be stated as**^1^**. It is calculated from equation 1.

$T_{S}= a_{f,i,pw}+b_{f,i}(T_{11}-T_{12})^{\frac{1}{\cos\left( \frac{\theta}{m} \right)}}+\left( b_{f,i}+c_{f,i} \right)T_{12}$ (1)

where Ts is LST in K; a, b, and c are coefficients; T11 and T12 are the BTs in S8 and S9, respectively; subscript f corresponds to vegetation fraction; i denotes vegetation type; pw is the CWVC; θ is the satellite zenith view angle; and m is a variable depending on θ.

**Appendix B. Calculating Landsat8 LST**

Thereby, the following steps were considered. First, radiometric calibration was used to extract the surface temperature from thermal bands, and digital numbers were converted into spectral radiation through the relationship 2.

$L\lambda=M_{L}*Q_{cal}+A_{L}$ (2)

ML and AL is the scaling factor and Qcal is the digital number in the considered band. After converting the digital number to reflection, the TIRS band data should be converted from spectral radiance to brightness temperature (BT) using thermal constants (Table 1). Equation 3 is used to convert spectral radiation to BT**^2^**.

$\mathrm{BT}=\frac{K_{2}}{\ln[\left( K_{1}/L\lambda\right)+1]} -273.15$ (3)

**Lλ** is the spectral radiation and **Ln** is the logarithm of Neprin and K1 and K2 are the specific thermal constants of the band obtained from the metadata file. To obtain results in Celsius, the radiant temperature is corrected by adding 273.15 °c**^3^**.

Table 1

Metadata of the satellite images.

|  | Thermal constant, Band 10 | |  | |
| --- | --- | --- | --- | --- |
| 1321.08  777.89 | K_1_  K_2_ | | | |
|  | Rescaling factor, Band 10 | |  | |
| 0.000342  0.1 | | M_L_  A_L_ | |  |

Considering that vegetation either plays a significant role in the energy transfer between the surface and the atmosphere or has different effects on climatic elements, including the surrounding temperature, can be one of the influential factors in LST calculation. In this regard, the NDVI index is representative of investigating vegetation. It is one of the most famous, simplest, and practical indices used in the study of vegetation. It not only has a pretty simple calculation process compared to other vegetation indices, but also has highly dynamic power. This index is most sensitive to vegetation changes and less sensitive to atmospheric and soil texture effects. Red (4) and near-infrared (5) bands of Landsat 8 have been used to estimate NDVI. The formula for calculating the NDVI index is calculated using equation 4.

$NDVI=\frac{(NIR-RED)}{(NIR+RED)}$ (4)

In the above relation, NIR is the near-infrared band, and RED is the red band. The value of this index changes from +1 to -1. Negative values are for areas without vegetation, and positive values are for areas with vegetation. Also, in this index, values less than zero are used for surfaces with water and clouds, and values between zero and 0.2 are used for bare soil. Values above 0.2 indicate vegetation density**^4^**. Considering the direct relationship between vegetation and land surface temperature, the positive value of this index is mainly associated with lower soil surface temperature and its negative value with higher surface temperature. In the following, equation 5 is used to determine the ratio of vegetation cover (P_V_), which indicates the vegetation cover ratio in an area.

$P_{v}=\left. \left( \frac{NDVI-NDVI_{s}}{\mathrm{NDV}I_{v}-NDVI_{s}} \right. \right)^{2}$ (5)

In this regard, NDVI values ​​greater than or equal to 0.5 (NDVIv) and between 0.2 and 0.5 (NDVIs) are used to show the general state of the vegetation ratio**^5^**. If NDVI is calculated from TOA reflectance, the values ​​of NDVIV and NDVI𝑠 will depend on atmospheric conditions**^6^**. Also, to estimate LST, it is necessary to calculate the emissivity of the land surface (LSE) based on the vegetation ratio (P_V_). Land surface emissivity (LSE) determines the amount of thermal energy transferred from the surface to the atmosphere**^7,8^**. LSE largely depends on surface roughness, the nature of vegetation, and soil**^9^**. Determining the emissivity is conditionally calculated from Equation 6**^5^**.

$\varepsilon_{\lambda}={\varepsilon_{v\lambda}P}_{v}+ \varepsilon_{s\lambda}(1-P_{v})+C_{\lambda}$ (6)

Ev and Es are the emission rates of vegetation and soil, respectively, and 𝐶 represents the surface roughness (the value of c is equal to zero for homogeneous and flat surfaces) which is considered as a constant value of 0.005. The amount of emission of different surfaces of the earth is calculated using equation 7.

$\varepsilon_{\lambda}=$

$\left\{ \begin{aligned} E_{s\lambda}\mathrm{NDVI}<NDVI_{s} \\ {\varepsilon_{v\lambda}P}_{v}+\varepsilon_{s\lambda}\left( 1-P_{v} \right)+C NDVI_{s} \leq NDVI \leq NDVI_{v} \\ \varepsilon_{s\lambda}+C NDVI>NDVI_{v} \end{aligned} \right.$ (7)

When NDVI is less than 0, it is classified as water and its emission value is 0.991. NDVI values between 0 and 0.2 are representative of land with soil and the emission value of 0.996 is assigned to it. Values between 0.2 and 0.5 indicate a combination of soil cover and vegetation, which equation (8) is used to calculate its diffusion capability. In the last case, when the NDVI value is greater than 0.5, vegetation is considered and its emission value is determined as 0.973. The last step is the calculation of LST or the corrected amount of radiation of the earth's surface temperature based on equation 8.

$T_{s}=\frac{\mathrm{BT}}{\{1+\left[ \left( \lambda\mathrm{BT}/\rho\right)\ln\varepsilon_{\lambda} \right]\}}$ (8)

Ts is the LST in degrees Celsius. BT is the BT at the sensor, λ is the average wavelength of band 10, ԑλ is the emissivity calculated in Equation 6, and ρ is calculated through Equation 9.

$\rho\boldsymbol{=}h\frac{c}{\sigma}=1.438\times{10}^{-2}mk$ (9)

σ is Boltzmann's constant (J/K10-23 x 1.38), h is Planck's constant (6.626 x 10-34), and c is the speed of light (m/s 108 x 3).

**Appendix C. Moran’s I**

In many parametric statistical methods (linear regression), it is assumed that the errors in the analysis are independent of each other and have no Correlation. When this assumption is not stated in the time series research project context, the errors become autocorrelated or dependent. Spatial autocorrelation is the correlation between the values of a variable that strictly refers to their relatively close locations on a two-dimensional surface. Since the phenomena of the natural world have order, pattern, and systematic focus, spatial autocorrelation exists. On the other hand, spatial autocorrelation means that the dependence between the values of a variable not only exists in nearby (neighboring) locations, but also has a systematic pattern through whole locations on the map. According to Tobler's first law of geography**^10^** "Everything is related to something else; however, this relationship is stronger in things that are closer compared to things that are far away"**^11^**.

Hence, autocorrelation is so important because it can affect the significance level (For example, positive autocorrelation leads to a decrease in p-value and significance level and, on the contrary, an increase in similarity). Also, the knowledge of its existence can guide the researcher to choose a more appropriate statistical analysis, and the accuracy of predictions using regression equations can be improved using autocorrelation information. Spatial autocorrelation theory has been a key element of geographic analysis for more than twenty years. Although there are various correlation tools, one of the most valuable (well-known) geographic statistics for detecting spatial autocorrelation is Moran's index**^12^**, a generalization of Pearson's correlation coefficient. Moran is divided into two categories, local and global, where local Moran is developed from global Moran. The local and global Moran describes the local clusters and the entire data set. Local Moran usually shows how each observation is similar to its neighbor by creating a single statistic, in the case which they are placed inside the clusters; otherwise, they are outside the cluster. One of the most important problems of using Moran's local index is the coincidence of possible values, which makes it difficult to reuse the same data in the conclusion process. Global indices of spatial autocorrelation are used to evaluate similar observed events close to each other**^13-16^**. Global Moran's statistic values range from +1 to -1; values greater than 0 indicate positive spatial autocorrelation (cluster pattern), values less than 0 indicate negative spatial autocorrelation (dispersed pattern), and 0 indicates the absence of spatial autocorrelation. (LST is randomly distributed). The higher the values, the greater the similarity of the values of the selected unit with the adjacent unit. The global Moran spatial autocorrelation is calculated using equation 10.

$I=\frac{n\sum_{j=1}^{n} {\sum_{j=1}^{n} w_{i,j}z}_{i}z_{j}}{S_{0}\sum_{i=1}^{n} z_{i}^{2}}$ (10)

Here, Zi is the difference between the feature value of complication i with its average (x-xi) and Wi is the spatial weight between complication i and j; n is the total number of geographic complications in the used layer and S0 is the sum of all spatial weights calculated through equation 11 is counted.

$S_{0}=\sum_{i=1}^{n} \sum_{j=1}^{n} w_{i,j}$ (11)

Standard Zia score for Moran's statistic is calculated through equation 12.

$z_{i}=\frac{I-E[I]}{\sqrt{V[I]}}$ (12)

where the values of E and V are obtained from relations 13 and 14:

(13) $E\left[ I \right]=-\frac{1}{n-1}$

(14) $V\left[ I \right]=E\left[ I^{2} \right]-E[I]^{2}$

**Appendix D. Getis- Ord G statistic**

Since Gi is a kind of Z score, it does not need any other calculations. The measurement of the Getis- Ord G statistic is in such a way that the positive values of this index that are statistically significant, and the larger they are, indicate proper clustering and the creation of hot spots (heat islands). The smaller the negative score of this statistically significant index means the clustering of low values will form cold spots. Also, each level in the Getis- Ord G statistic has a z-score. Higher positive z values ​​were classified as hot spots and smaller negative z values ​​as cold spots. Z-value indicates the importance of clustering for a specific interval based on the significance level**^17^**.

Analysis of hot spots, the Getis- Ord G statistic is calculated from equation 15.

$G_{j}=\frac{\sum_{j=1}^{n} w_{i,j}{wX}_{j}- \bar{X}\sum_{j=1}^{n} w_{i,j}}{\sqrt[S]{\frac{n[n\sum_{j=1}^{n} w_{i,j}^{2}-(\sum_{j=1}^{n} w_{i,j})^{2}-1}{n-1}}}$ (15)

In this formula, Xj is the attribute value for complication j, Wij, the spatial weight between complications i, j and n is equal to the total number of complications. How to calculate X ̅ and S is given in equations 16 and 17.

(16) $(\bar{X}=\frac{\sum_{j=1}^{n} X_{j}}{n}$

(17) $s=\sqrt{\frac{\sum_{j=1}^{n} X_{j}^{2}}{n}}-(\bar{X})^{2}$

**References**

1. Remedios J, Emsley S. Sentinel-3 Optical Products and Algorithm Definition Land Surface Temperature. 24
2. USGS, 2013, [http://landsat.usgs.gov/Landsat8 Using Product .php](http://landsat.usgs.gov/Landsat8%20Using%20Product%20.php). (2012).
3. Xu, H., Chen, B. Remote sensing of the urban heat island and its changes in Xiamen City of SE China. Journal of environmental sciences, 16 2, 276-81. (2004).
4. Akbar, T. A., Hassan, Q. K., Ishaq, S., Batool, M., Butt, H. J., Jabbar, H. Investigative Spatial Distribution and modelling of existing and future urban land changes and its impact on urbanization and economy. Remote Sensing, 11(2), 105. https://doi.org/10.3390/rs11020105 (2019).
5. Sobrino, J.A., Jim´enez-Mu˜noz, J.C., Paolini, L. Land surface temperature retrieval fromLANDSAT TM5, Remote Sensing of Environment, 90 (4), 434–440. (2004).
6. Jimenez-Munoz, J., Sobrino, J., Plaza, A., Guanter, L., Moreno, J., Martinez, P. Comparison Between Fractional Vegetation Cover Retrievals from Vegetation Indices and Spectral Mixture Analysis: Case Study of PROBA/CHRIS Data Over an Agricultural Area. Sensors, 9(2), 768-793. (2009).
7. Jimenez-Munoz, J.C., Sobrino, J.A., Gillespie, A., Sabol, D., Gustafson, W. Improved land surface emissivities over agricultural areas using ASTER NDVI. Remote Sensing Of Environment, 103(4), 474-487. (2006).
8. Avdan, U., Jovanovska, G. Algorithm for Automated Mapping of Land Surface Temperature Using LANDSAT 8 Satellite Data. Journal Of Sensors, 2016, 1-8. https://doi.org/10.1155/2016/1480307 (2016).
9. Mallick, Javed. Kant., Yogesh., Bharath., B.D. Estimation of Land Surface Temperature over Delhi Using Landsat-7 ETM+, J. Ind. Geophys. Union, 12 (3), 131-140. (2008).
10. Tobler, W. R. A computer movie simulating urban growth in the Detroit region. Economic Geography, 46(sup1), 234–240. (1970).
11. Griffith, D.A. Spatial Autocorrelation. International Encyclopedia of Human Geography. 308-316. https://doi.org/10.1016/B978-008044910-4.00522-8 (2009).
12. Moran PAP . The interpretation of statistical maps. Journal of the Royal Statistical Society, Series B, 37(2): 243-251. (1948).
13. Walter SD. The analysis of regional patterns in health data. II. The power to detect environmental effects. Am J Epidemiol, 136(6):742-759. (1992).
14. Rogerson P. The detection of clusters using a spatial version of the ChiSquare Goodness-of-Fit Statistic. Geographical Analysis, 31(1):128-147. (1999).
15. Waller LA, Gotway CA. Applied Statistics for Public Health Data. New York: Wiley. (2004).
16. Jackson MC, Waller LA. Exploring Goodness-of-fit and spatial correlation using components of Tango's Index of spatial clustering. Geographical Analysis, 37(4):371-382. (2005).
17. ESRI. What is a z-Score? What is a p-Value? 2016. Available online: http://pro.arcgis.com/en/pro-app/ tool-reference/spatial-statistics/what-is-a-z-score-what-is-a-p-value.htm (2016).
